# XPC-RAD23B enhances UV-DDB binding to DNA to facilitate lesion search in nucleotide excision repair

**DOI:** 10.1093/nar/gkaf463

**Published:** 2025-06-18

**Authors:** Soyeong An, Masayuki Kusakabe, Hyun-Suk Kim, Hidetsugu Kozono, Na Young Cheon, Jeongeun Kim, Jieun Kang, Sunbok Jang, Kaoru Sugasawa, Orlando D Schärer, Ja Yil Lee

**Affiliations:** Department of Biological Sciences, Ulsan National Institute of Science and Technology, Ulsan 44919, Republic of Korea; Biosignal Research Center and Graduate School of Science, Kobe University, Kobe, Hyogo 657-8501, Japan; Institute of Basic Science Center for Genomic Integrity, Ulsan 44919, Republic of Korea; Biosignal Research Center and Graduate School of Science, Kobe University, Kobe, Hyogo 657-8501, Japan; Department of Biological Sciences, Ulsan National Institute of Science and Technology, Ulsan 44919, Republic of Korea; College of Pharmacy, Graduate School of Pharmaceutical Sciences, Ewha Womans University, Seoul 03760, Republic of Korea; Graduate Program in Innovative Biomaterials Convergence, Ewha Womans University, Seoul 03760, Republic of Korea; College of Pharmacy, Graduate School of Pharmaceutical Sciences, Ewha Womans University, Seoul 03760, Republic of Korea; College of Pharmacy, Graduate School of Pharmaceutical Sciences, Ewha Womans University, Seoul 03760, Republic of Korea; Graduate Program in Innovative Biomaterials Convergence, Ewha Womans University, Seoul 03760, Republic of Korea; Biosignal Research Center and Graduate School of Science, Kobe University, Kobe, Hyogo 657-8501, Japan; Department of Biological Sciences, Ulsan National Institute of Science and Technology, Ulsan 44919, Republic of Korea; Institute of Basic Science Center for Genomic Integrity, Ulsan 44919, Republic of Korea; Graduate School of Health Science and Technology, Ulsan National Institute of Science and Technology, Ulsan 44919, Republic of Korea; Department of Pharmacology and Chemical Biology & Hillman Cancer Center, University of Pittsburgh Medical School, Pittsburgh, PA 15213, United States; Department of Biological Sciences, Ulsan National Institute of Science and Technology, Ulsan 44919, Republic of Korea; Institute of Basic Science Center for Genomic Integrity, Ulsan 44919, Republic of Korea

## Abstract

Ultraviolet-induced DNA lesions are removed by the nucleotide excision repair (NER) pathway. In global-genome NER (GG-NER), XPC-RAD23B recognizes the lesions and initiates NER. However, cyclobutane pyrimidine dimers (CPDs), which do not significantly destabilize the DNA duplex, are not bound by XPC-RAD23B with high selectivity. Instead, CPD is preferentially sensed by UV-DDB, which is believed to hand over the lesion to XPC-RAD23B via ubiquitination of both proteins. Here, by combining biochemical and single-molecule DNA curtain assays, we investigate the interactions between UV-DDB and XPC-RAD23B on DNA. Surprisingly, we discover that XPC-RAD23B enhances the binding of UV-DDB to DNA. We demonstrate that this enhancement can be attributed to the complex formation of UV-DDB and XPC-RAD23B (UX-complex), which increases the binding affinity of UV-DDB to undamaged DNA. We further show that UV-DDB finds CPDs through one-dimensional (1D) diffusion along DNA. Collectively, the UX-complex enhances UV-DDB loading to DNA to accelerate the search for CPD via 1D diffusion. Moreover, we find that UV-DDB and XPC-RAD23B can bind CPDs as a complex, which facilitates the transfer of CPD. Altogether, our results show that UV-DDB and XPC-RAD23B cooperatively interact for rapid CPD search, providing a new mechanism for lesion search in GG-NER.

## Graphical Abstract

**Graphical Abstract:**
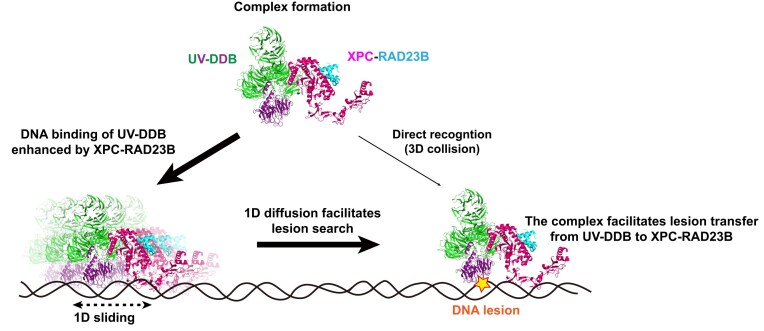

## Introduction

Ultraviolet (UV) light induces DNA modifications such as cyclobutane pyrimidine dimer (CPD) and pyrimidine (6-4) pyrimidone photoproduct (6-4PP) [1, 2]. The UV lesions are removed from DNA by the nucleotide excision repair (NER) pathway [2, 3]. Defects in NER cause xeroderma pigmentosum (XP) characterized by extreme sensitivity to sunlight exposure and predisposition to skin cancer [4]. NER also repairs a broad range of DNA lesions caused by cancer therapeutics such as cisplatin and environmental mutagens that cause bulky chemical adducts [4]. There are two NER pathways that differ in their mechanism of lesion detection. In transcription-coupled NER (TC-NER), a transcribing RNA polymerase is stalled at a DNA lesion and serves as a marker for DNA damage [5]. In global-genome NER (GG-NER), DNA lesions are recognized by the XP complementation group C (XPC) protein that stably associates with RAD23B. XPC-RAD23B senses helical destabilization of DNA caused by the damage [3, 6] and binds to the strand opposite to the lesion. Thus, it can identify diverse types of DNA damage [7]. Subsequently, TFIIH, XPA, and RPA join XPC-RAD23B at the lesion to form the pre-incision complex. TFIIH verifies whether or not the lesion can be repaired by NER [3]. After the verification, XPF-ERCC1 and XPG endonucleases join the pre-incision complex and make incisions around the lesion [3]. A lesion-containing oligonucleotide is removed, and the gap is filled by DNA polymerases. Then the nick is sealed by DNA ligases to finally complete the repair [3, 8].

**Figure 1.**
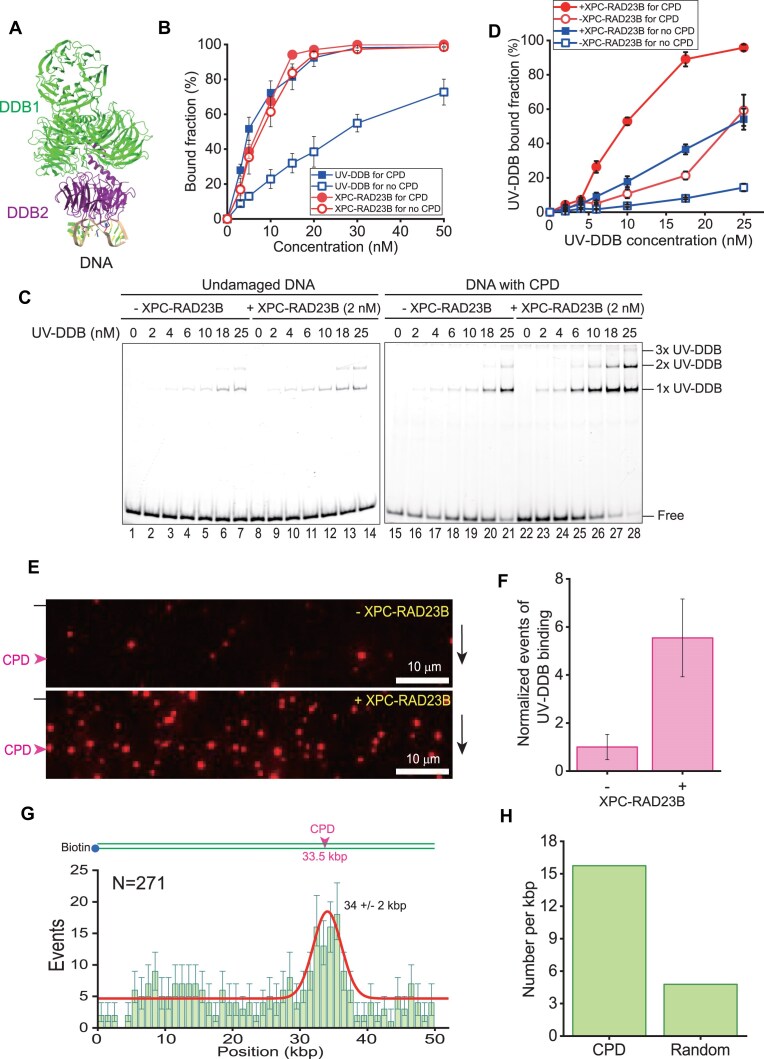
XPC-RAD23B enhances UV-DDB binding to DNA. (**A**) The structure of UV-DDB bound to damaged DNA (PDB: 4a08) [12]. (**B**) Binding affinity of UV-DDB and XPC-RAD23B to undamaged and CPD-containing DNA. Quantification of electrophoretic mobility shift assay (EMSA) in Supplementary Fig. S1E, where UV-DDB and XPC-RAD23B are titrated (0, 3, 5, 10, 15, 20, 30, and 50 nM) to 4 nM undamaged and CPD-containing DNA in the absence of competitors. Error bars are obtained from standard error in triplicate. (**C**) EMSA for UV-DDB titration (0, 2, 4, 6, 10, 18, and 25 nM) to 10 nM undamaged DNA (left) and CPD-containing DNA (right) in the absence and presence of 2 nM XPC-RAD23B. (**D**) Quantification of EMSA in panel (C). Error bars are obtained from standard error in triplicate. (**E**) Fluorescence images of 0.2 nM Qdot-labeled UV-DDB in the DNA curtains in the absence (top) and presence (bottom) of 0.4 nM XPC-RAD23B under the buffer flow. The black bar and magenta arrowhead at the left represent the barrier and CPD positions, respectively. The black arrow at the right indicates the flow direction. (**F**) Total binding number of UV-DDB molecules in the DNA curtains depending on XPC-RAD23B. The number of DNA-bound UV-DDB is estimated by counting the number of fluorescent puncta in the DNA curtains. For the normalization, the number of DNA-bound UV-DDB in the presence of XPC-RAD23B is divided by that in the absence of XPC-RAD23B. Error bars are obtained from standard error in triplicate. (**G**) (Top) Lambda phage DNA (λ-DNA) construct containing CPD sites around 33.5 kbp. (Bottom) Binding position distribution of 0.2 nM UV-DDB in the presence of 0.4 nM XPC-RAD23B. Error bars are obtained from bootstrapping with 70% confidence interval. The histogram is fitted with a single Gaussian function, which is centered at 34 ± 2 kbp. *N* indicates the total number of analyzed molecules. (**H**) Number of UV-DDB molecules per unit length (kbp). The number bound to CPD and random sites is estimated as the molecule counts between 32 and 36 kbp and placed at the other sites, respectively. The number is then divided by the length.

Although XPC-RAD23B can identify various types of DNA damage including photolesions, it is inefficient in finding CPDs, as CPD only weakly destabilizes the DNA duplex [9, 10]. Therefore, another protein, UV-DDB, is necessary for sensing CPDs. UV-DDB is a heterodimer consisting of DDB1 and DDB2 (Fig. 1A) [11–13]. It binds CPD as well as 6-4PP with high affinity [14, 15]. In the absence of DDB2, CPDs are barely repaired in cells [16]. Mutations in DDB2 cause the XP complementation group E, which is characterized by defects in GG-NER like mutations in XPC but not in TC-NER [17]. UV-DDB has an important role in finding DNA lesions in chromatin [18, 19]. UV-DDB forms a complex with Cullin4A E3 ubiquitin ligase [20]. After binding a lesion, the UV-DDB–Cullin4A complex ubiquitinates histones to remodel compact chromatin for the recruitment and assembly of downstream NER proteins at the lesion [21]. UV-DDB–Cullin4A also ubiquitinates DDB2 and XPC-RAD23B, and this process is needed for the handover of the lesion from UV-DDB to XPC-RAD23B [22]. Ubiquitinated UV-DDB dissociates from the lesion, while XPC-RAD23B engages with the lesion [23].

Single-molecule tightrope assays showed that wild-type UV-DDB finds UV-induced DNA lesions through three-dimensional (3D) collision without diffusion along DNA [24]. In contrast, DDB2 containing the XP-causing mutation K244E diffused along DNA without stable binding to UV lesions. When UV-DDB encounters UV lesions, a β-hairpin in the WD40 domain of DDB2 is inserted into the minor groove and flips the lesion into a binding pocket accompanied by a kink of DNA [12]. Even though UV lesion search and recognition by UV-DDB have been studied, how UV-DDB and XPC-RAD23B collaborate for lesion search, recognition, and transfer remains poorly understood.

Here, using single-molecule imaging and biochemical assays, we discovered that XPC-RAD23B promotes CPD search of UV-DDB, which we attribute to a complex formation of UV-DDB and XPC-RAD23B. Fluorescence recovery after photobleaching (FRAP) assay provides evidence that this complex is formed inside cells. We attribute the binding enhancement of UV-DDB by XPC-RAD23B to the difference in binding affinity to CPDs and undamaged DNA of UV-DDB and XPC-RAD23B. Surprisingly, our single-molecule imaging demonstrated that wild-type UV-DDB diffuses along DNA to search for DNA lesions in addition to 3D collision to CPD. Taken together, our findings suggest that UV-DDB and XPC-RAD23B cooperate to facilitate CPD recognition and repair in NER.

## Materials and methods

The details of materials and methods are described in the Supplementary data.

### Protein purification

All proteins were purified at 4°C.

Purification of human UV-DDB with 1× FLAG, PreScission protease cleavage sequence, and 3× HA tags at the amino-terminus was followed by the previous protocol with slight modifications [22]. UV-DDB was overexpressed in High-Five insect cells. The cells were lysed by sonication in 80 ml of lysis buffer [50 mM Tris–HCl (pH 8.0), 1 mM EDTA, 0.5 M NaCl, 0.25 mM TCEP, 10% glycerol, and Halt protease inhibitor cocktail (Thermo Fisher Scientific, 78438)]. UV-DDB was purified by anti-FLAG M2 agarose beads (Merck, A2220). The eluates were treated with 8.5 μg/ml of PreScission protease overnight to remove 1× FLAG from the amino terminus. The proteins were then purified using a HiTrap heparin HP column (1 ml, Cytiva, 17040601) and MonoQ column (Cytiva, 29275878) by salt gradient, successively. The purified proteins were stored at −80°C until use. The activity of purified UV-DDB was tested by a pull-down assay with UV-damaged DNA.

Purification of human XPC-RAD23B was followed by the previous purification protocol [25]. Briefly described, XPC-RAD23B with 3× FLAG at amino-terminus of XPC was overexpressed in 0.4 l of insect cells (Sf9). The resuspended cells were lysed by a Dounce homogenizer and then clarified by centrifugation (40 000 × *g* for 30 min). The clarified lysates were purified with anti-FLAG M2 agarose beads. The proteins were subsequently purified through a gel filtration column (HiLoad 16/600 Superdex 200, Pharmacia). XPC-RAD23B was further purified and concentrated by heparin column (1 ml of HiTrap Heparin, Pharmacia) with NaCl gradient from 0.2 to 1.5 M. The eluates were then dialyzed overnight against storage buffer [25 mM potassium phosphate (pH 7.6), 100 mM NaCl, 10% glycerol, and 5 mM 2-mercaptoethanol (2ME)] and then stored at −80°C after snap-freezing in liquid nitrogen. The activity of purified XPC-RAD23B was tested by *in vitro* NER assay [25, 26].

### DNA curtain assays

Single-tethered and double-tethered DNA curtain experiments were performed as described before [25, 27, 28]. CPD-containing lambda phage DNA (λ-DNA) was used for damage search mechanism of UV-DDB (Supplementary Fig. S2A and B). UV-DDB was labeled with fluorescent nanoparticle, quantum dot (Qdot), which was conjugated with anti-HA antibodies (SiteClick Qdot Antibody Labeling Kits, Thermo Fisher Scientific, S10454).

### Electrophoretic mobility shift assay

All reactions were performed in reaction buffer [50 mM Tris–HCl (pH 7.5), 100 mM NaCl, 0.1 mg/ml bovine serum albumin (BSA), and 1 mM dithiothreitol (DTT)] at 23°C. For the binding affinity of either UV-DDB or XPC-RAD23B, 4 nM annealed DNA was incubated with each protein at different concentrations in reaction buffer at 23°C for 45 min in a dark room to prevent photobleaching of FAM. For the EMSA with competitors, 200 nM unlabeled DNA oligomers (50×) were simultaneously added to the reactant. Except that, all other EMSA experiments were conducted with 10 nM DNA under the same condition. The reactions were analyzed by 6% non-denaturing polyacrylamide gel electrophoresis (PAGE), and the gel was imaged by Typhoon RGB (Cytiva).

### Native gel western blot

To check XPC-RAD23B and UV-DDB complex, we performed native gel western blot. For this, the 6% PAGE was run for longer time (about 3 h) to spread each band. The gel was imaged by Typhoon RGB to check band position on the gel. The shifted bands on the gel were transferred to the membrane to perform western blot using HA antibody (1:10 000) (Invitrogen, 26183) and XPC antibody (1:300) (SantaCruz, sc-74410).

### Immunoprecipitation

*In vitro* immunoprecipitation (IP) was conducted using anti-FLAG M2 agarose beads. 50 nM XPC-RAD23B was incubated with anti-FLAG M2 agarose beads at 4°C overnight. After unbound XPC-RAD23B was washed out, the XPC-RAD23B bound beads were incubated with 100 nM UV-DDB in the presence or absence of 1 μg/ml DNase I (Merck, D5319-2) in 30 μl of total reaction volume with NETN buffer [20 mM Tris–HCl (pH 8.0), 100 mM NaCl, 1 mM EDTA, 0.1% NP-40] with 0.1 mg/ml BSA at 4°C for 1 h. After washing with NETN buffer, the eluates were checked by western blot with anti-XPC antibody (1:300) (SantaCruz, sc-74410) and anti-HA antibody (1:10 000) (Invitrogen, 26183).

### Fluorescence recovery after photobleaching

U2OS cells having stable expression of DDB2-mKO1 were used, in which endogenous DDB2 gene or both endogenous DDB2 and XPC genes were disrupted. Cells were irradiated with UV-C at different doses (0, 1, or 10 J/m^2^). FRAP was performed within 30 min after UV-C irradiation. Under the confocal laser microscope system (Evident, FV3000), approximately a half area of nucleus was bleached with the 561 nm laser at 100% power. After photobleaching, the fluorescence images were acquired every 1 s for 1 min, and data analyses were performed as described previously [29].

### Surface plasmon resonance assay

All surface plasmon resonance (SPR) experiments were performed using a Nicoya OpenSPR XT rev4 instrument (Nicoya Lifesciences) using a carboxyl-functionalized sensor chip. All experiments were conducted at 25°C, and all solutions were filtered (0.22 μm) and degassed before use. Prior to immobilization, the chip surface was activated by injecting a 1:1 mixture of 100 mM EDC (1-ethyl-3-(3-dimethylaminopropyl)carbodiimide hydrochloride) and 100 mM NHS (N-hydroxysuccinimide) for 600 s at a flow rate of 10 μl/min. UV-DDB (ligand) was diluted to 21 μg/ml in 10 mM sodium acetate buffer (pH 4.5) and immobilized via amine coupling by injection for 600 s at 10 μl/min, resulting in a final immobilization level of ∼2500 response units (RUs). Residual reactive esters were quenched by injecting 1 M ethanolamine–HCl (pH 8.5) for 300 s. To minimize nonspecific binding, 1 mg/ml BSA diluted in 10 mM sodium acetate buffer (pH 4.5) was injected over both ligand and reference channels for 600 s. The reference channel underwent identical activation and blocking steps, without UV-DDB injection. XPC-RAD23B (analyte) was diluted in running buffer [10 mM potassium phosphate (pH 7.5) and 150 mM NaCl] to different concentrations (10, 25, 50, 100, 200, 400, and 600 nM) and injected over the sensor surface at 20 μl/min for 300 s, followed by a dissociation phase of 600 s. After each analyte injection, the surface was regenerated with a 30-s injection of 10 mM glycine–HCl (pH 1.5). The SPR assays were conducted at each analyte concentration in triplicate. Sensorgrams were processed and corrected by subtracting the signal from the reference channel. Binding responses were analyzed using steady-state affinity models.

## Results

### XPC-RAD23B enhances the binding of UV-DDB to DNA

To investigate the interaction of UV-DDB or XPC-RAD23B with native and damaged DNA, we conducted EMSA using purified proteins (Fig. 1B and Supplementary Fig. S1). As shown previously, UV-DDB exhibited preferential binding for CPD-containing DNA [14, 15]. Consistent with previous studies, UV-DDB bound to CPDs with about five times higher binding affinity (*K*_d_ ∼ 5 nM) compared to undamaged DNA (*K*_d_ ∼ 26 nM) (Fig. 1B and Supplementary Fig. S1E) [30, 31]. By contrast, XPC-RAD23B had similar binding affinity for CPD-containing DNA (*K*_d_ ∼ 7 nM) and undamaged DNA (*K*_d_ ∼ 9 nM), in the range of the binding affinity of UV-DDB to CPD (Fig. 1B and Supplementary Fig. S1E). Interestingly, the binding affinity of XPC-RAD23B for undamaged DNA was about three times higher than that of UV-DDB. Therefore, XPC-RAD23B barely distinguished CPDs from homoduplex DNA as previously reported [9]. We further investigated the binding specificity of UV-DDB and XPC-RAD23B to CPDs in the presence of excess competitor (Supplementary Fig. S1F and G). There was little change in the binding affinity of UV-DDB to CPDs in the presence of competitors, while the binding affinity to undamaged DNA was significantly reduced, showing that UV-DDB exhibits considerable specificity for binding to CPDs. The addition of competitors dramatically reduced the binding affinity of XPC-RAD23B to both CPD and homoduplex even though the protein showed higher binding affinity to CPD in the presence of competitors, in line with a weak preference of XPC-RAD23B for CPDs over homoduplex DNA. These results were consistent with previous studies [9, 32].

We next investigated the interaction between UV-DDB and XPC-RAD23B and conducted EMSA for UV-DDB in the presence of 2 nM XPC-RAD23B, a concentration at which XPC-RAD23B did not exhibit detectable binding to either undamaged or CPD-containing DNA (lane 8 and lane 22 in Fig. 1C). In the presence of 2 nM XPC-RAD23B, the intensity of UV-DDB–DNA bands was increased, indicating that XPC-RAD23B stimulates the binding of UV-DDB to both undamaged and CPD-containing DNA. Quantitatively, the addition of XPC-RAD23B increased the binding affinity of UV-DDB to CPD by 2.5 times (Fig. 1D). Of note, the UV-DDB binding was enhanced even at UV-DDB concentrations higher than XPC-RAD23B concentration (2 nM), implying that XPC-RAD23B would repeatedly interact with UV-DDB to promote the binding of UV-DDB to DNA.

To obtain further evidence for this mechanism, we visualized UV-DDB binding to DNA using single-tethered DNA curtain assays with 3× CPD-containing λ-DNA (Supplementary Fig. S2). In the single-tethered DNA curtain experiment, only one end of λ-DNA was anchored on the lipid bilayer, and hence λ-DNA molecules were stretched at nano-trenches under buffer flow [28]. UV-DDB was labeled through its 3× HA tag at the N-terminus of DDB1 subunit with anti-HA-conjugated quantum dots (Qdots). In the absence of XPC-RAD23B, UV-DDB rarely bound to DNA at a concentration of 0.2 nM, whereas the number of bound UV-DDB molecules was increased by co-injection of 0.4 nM XPC-RAD23B (Fig. 1E). Quantitatively, co-injection with XPC-RAD23B stimulated UV-DDB binding to DNA by 5.5-fold (Fig. 1F). The binding distribution of UV-DDB in the presence of XPC-RAD23B displayed a distinct single peak around the 3× CPD sites, indicating the preferential binding of UV-DDB to CPD (Fig. 1G). When the number of bound UV-DDB molecules per unit length (kbp) was estimated from the distribution, the binding at CPD sites was increased three-fold compared to random sequences, suggesting that XPC-RAD23B not only increases DNA loading but also promotes CPD recognition of UV-DDB (Fig. 1H). Taken together, our biochemical and single-molecule results show that XPC-RAD23B enhances the binding of UV-DDB to DNA.

### UV-DDB and XPC-RAD23B can simultaneously bind to CPDs

**Figure 2.**
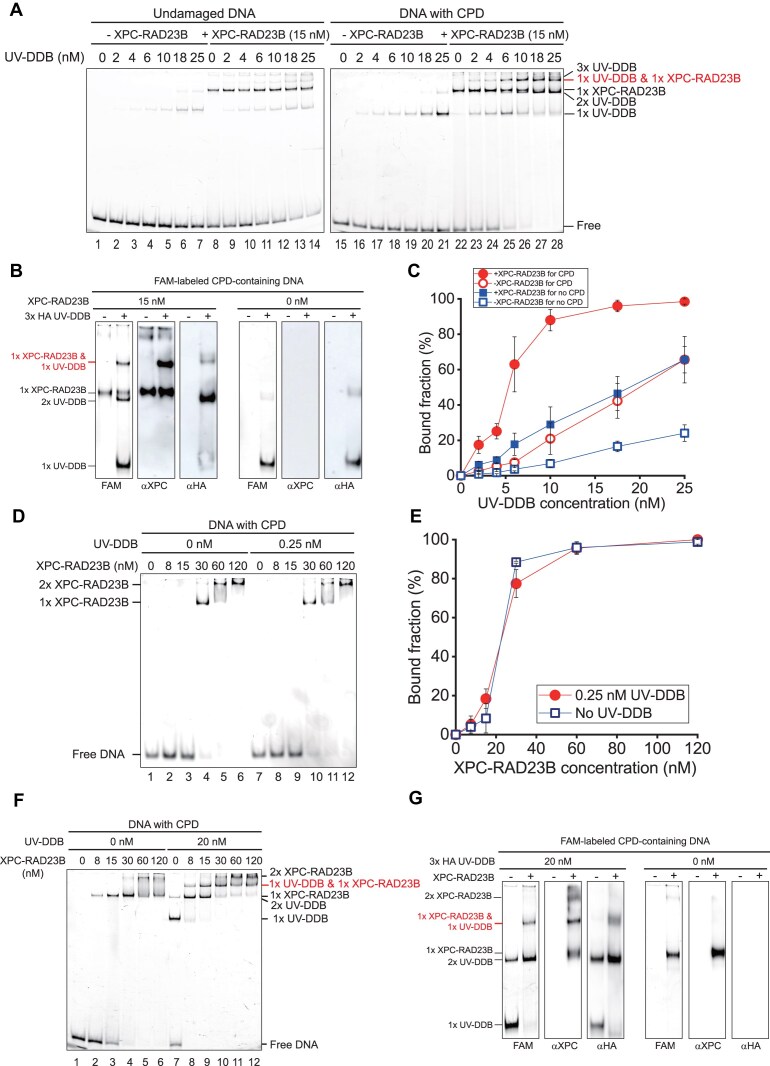
Co-binding of XPC-RAD23B and UV-DDB. (**A**) EMSA for UV-DDB binding (0, 2, 4, 6, 10, 18, and 25 nM) to undamaged (left) and CPD-containing DNA (right) in the absence and presence of 15 nM XPC-RAD23B. (**B**) Western blot assay with the native EMSA gel for 10 nM UV-DDB with CPD-containing DNA in the presence and absence of 15 nM XPC-RAD23B. The gels are blotted with indicated antibodies. (**C**) Quantification of EMSA in panel (A). Error bars are obtained from standard error in triplicate. (**D**) EMSA for XPC-RAD23B titration (0, 8, 15, 30, 60, and 120 nM) to CPD-containing DNA in the absence and presence of 0.25 nM UV-DDB. (**E**) Quantification of EMSA in panel (D). Error bars are obtained from standard error in triplicate. (**F**) EMSA for XPC-RAD23B titration (0, 8, 15, 30, 60, and 120 nM) to CPD-containing DNA in the absence and presence of 20 nM UV-DDB. (**G**) Western blot assay with the native EMSA gel for 30 nM XPC-RAD23B with CPD-containing DNA in the presence and absence of 20 nM UV-DDB. The gels are blotted with indicated antibodies.

We then investigated the enhancement of DNA binding of UV-DDB by XPC-RAD23B in more detail. We conducted EMSA at a higher XPC-RAD23B concentration (15 nM), at which XPC-RAD23B also bound to DNA (lane 8 and lane 22 in Fig. 2A). In the presence of 15 nM XPC-RAD23B, UV-DDB-bound bands appeared at lower UV-DDB concentrations than in the absence of XPC-RAD23B, consistent with our results showing that 2 nM XPC-RAD23B enhances UV-DDB binding to DNA (Fig. 1C and D). At 15 nM XPC-RAD23B, the binding enhancement was more prominent for CPD-containing DNA than for undamaged DNA (Fig. 2A). The bands for single DNA-bound UV-DDB (1× UV-DDB) and XPC-RAD23B (1× XPC-RAD23B) were clearly defined (lane 4 and lane 18 for UV-DDB, and lane 8 and lane 22 for XPC-RAD23B in Fig. 2A). With increasing UV-DDB concentration, the 1× UV-DDB and 1× XPC-RAD23B bands faded, while additional bands with lower mobility emerged. The band very close to the 1× XPC-RAD23B band could be assigned as two molecules of UV-DDB binding (2× UV-DDB) based on UV-DDB titration without XPC-RAD23B (lane 21 in Fig. 2A). Crystal structures of UV-DDB had previously shown that the protein can bind DNA as a dimer [13, 24]. For CPD-containing DNA, the top band at the highest UV-DDB concentration (lane 28 in Fig. 2A) was consistent with the binding of at least three UV-DDB molecules (3× UV-DDB, lane 28 of Fig. 1C). Unexpectedly, we observed a new band between 1× XPC-RAD23B and 3× UV-DDB bands. Because no band corresponding to an XPC-RAD23B dimer was observed, we surmised that the new band results from the binding of one molecule of each protein to CPD-containing DNA. To ascertain this possibility, we conducted western blot assays of the native EMSA gel (Fig. 2B). The bands were identified with either XPC or HA (for DDB1) antibodies, showing a signal for both proteins and verifying the presence of UV-DDB and XPC-RAD23B in this CPD-containing DNA band. Quantitatively, 15 nM XPC-RAD23B enhanced the UV-DDB binding by approximately four-fold compared to no XPC-RAD23B (Fig. 2C). These results demonstrated that XPC-RAD23B enhances UV-DDB binding to CPDs and the two proteins can simultaneously bind to CPD-containing DNA. To the best of our knowledge, this is the first demonstration of the simultaneous binding of UV-DDB and XPC-RAD23B to DNA containing a lesion.

### UV-DDB does not promote the binding of XPC-RAD23B to DNA

We next asked whether UV-DDB could also enhance the binding of XPC-RAD23B to DNA. We titrated XPC-RAD23B for CPD-containing DNA in the absence and presence of UV-DDB. However, there was no significant difference in binding affinity of XPC-RAD23B upon incubation with 0.25 or 2.5 nM UV-DDB (Fig. 2D and E, and Supplementary Fig. S3A and B). On the other hand, as the XPC-RAD23B concentration increased, the intensity of the 1× UV-DDB band increased, supporting the finding that UV-DDB binding is enhanced by XPC-RAD23B (lane 8 and lane 9 in Supplementary Fig. S3A). Moreover, another band emerged between 1× and 2× XPC-RAD23B bands (Supplementary Fig. S3A). Upon increasing the concentration of UV-DDB to 20 nM, the band became more prominent (Fig. 2F). As in Fig. 2B, the analysis of the native gel by western blot assay demonstrated that the band represents the simultaneous binding of UV-DDB and XPC-RAD23B to CPD (Fig. 2G). Collectively, our data demonstrated that UV-DDB does not enhance the binding of XPC-RAD23B to DNA.

### Complex formation of UV-DDB and XPC-RAD23B in solution promotes UV-DDB binding

**Figure 3.**
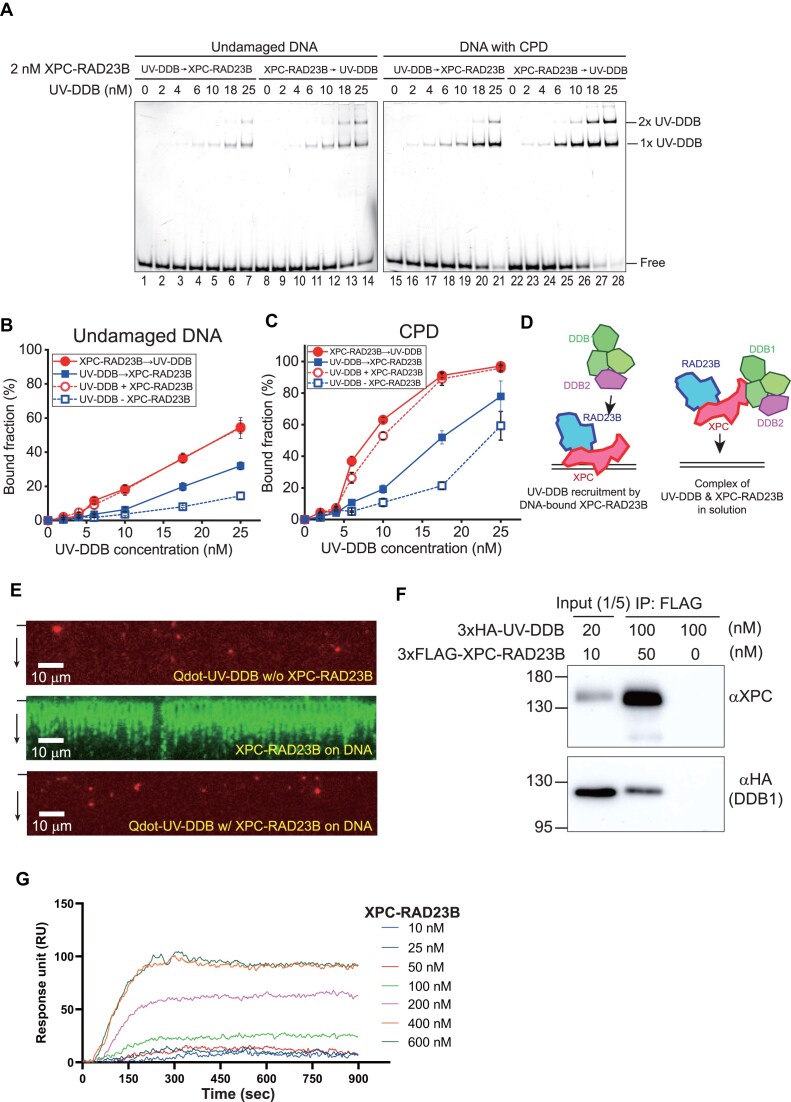
Enhancement mechanism: recruitment of UV-DDB by DNA-bound XPC-RAD23B or complex formation in solution. (**A**) EMSA for UV-DDB titration (0, 2, 4, 6, 10, 18, and 25 nM) to undamaged (left) and CPD-containing DNA (right) in the presence of 2 nM XPC-RAD23B. In this experiment, UV-DDB is incubated with DNA prior to XPC-RAD23B or vice versa. (**B**) Quantification of EMSA for undamaged DNA in panel (A). Error bars are obtained from standard error in triplicate. (**C**) Quantification of EMSA for CPD-containing DNA in panel (A). Error bars are obtained from standard error in triplicate. (**D**) Possible models for UV-DDB binding enhancement to DNA by XPC-RAD23B. (Left) DNA-bound XPC-RAD23B recruits UV-DDB and (right) the complex formation of UV-DDB and XPC-RAD23B has higher binding affinity to DNA. (**E**) DNA curtain images of 0.2 nM Qdot-labeled UV-DDB binding to DNA (top) in the absence and (middle and bottom) the presence of XPC-RAD23B. (Top) DNA curtain image for UV-DDB without XPC-RAD23B. (Middle) DNA curtain image for 8 nM XPC-RAD23B, which is incubated with DNA curtains and then labeled with Alexa488-conjugated FLAG antibody after unbound XPC-RAD23B is washed out. (Bottom) DNA curtain image for UV-DDB, which is incubated with DNA covered with XPC-RAD23B in the middle panel. The black bar and arrow represent the barrier position and buffer direction, respectively. (**F**) *In vitro* IP assay for the complex formation of UV-DDB and XPC-RAD23B. 3× HA-tagged UV-DDB is pulled down by 3× FLAG-tagged XPC-RAD23B, which is conjugated with anti-FLAG beads. The proteins are blotted by denoted antibodies. (**G**) SPR assay for the complex formation of UV-DDB and XPC-RAD23B. UV-DDB is immobilized on the surface of a SPR sensor chip via EDC and NHS. Different concentrations (10, 25, 50, 100, 200, 400, and 600 nM) of XPC-RAD23B were introduced at 20 μl/min for 300 s, followed by a dissociation phase of 600 s. The RUs are calculated by signal subtraction between the ligand channel containing UV-DDB and the reference channel without UV-DDB.

We further investigated the mechanism by which only XPC-RAD23B promotes the binding of UV-DDB to undamaged and CPD-containing DNA. To this end, we sequentially incubated UV-DDB and XPC-RAD23B with DNA or vice versa. When XPC-RAD23B was incubated with DNA before adding UV-DDB, the binding enhancement of UV-DDB was very similar to that in the co-incubation results (Fig. 3A–C). However, when UV-DDB was incubated with the DNA before addition of XPC-RAD23B, the binding was not enhanced to the same extent (Fig. 3A–C). The incubation of UV-DDB and DNA prior to XPC-RAD23B led to the binding of UV-DDB to DNA. Hence, these results suggested that XPC-RAD23B may not act on UV-DDB that is already bound to DNA. Given these results along with the concurrent binding of UV-DDB and XPC-RAD23B to CPD, we considered two possibilities for the DNA binding enhancement of UV-DDB by XPC-RAD23B: one was that DNA-bound XPC-RAD23B recruits UV-DDB to DNA and the other was that UV-DDB and XPC-RAD23B form a complex (UX-complex) in solution, which has higher binding affinity to DNA (Fig. 3D). The first possibility was tested using DNA curtain assays, in which XPC-RAD23B was bound to λ-DNA at high density and free XPC-RAD23B was washed out. Subsequently, 0.2 nM UV-DDB, which did not bind DNA molecules without XPC-RAD23B under these conditions (Fig. 1E), was injected. If DNA-bound XPC-RAD23B recruited UV-DDB, we would expect UV-DDB to be loaded onto λ-DNA even at the low concentration as shown in Fig. 1E. However, UV-DDB did not bind to λ-DNA covered with XPC-RAD23B, which had ∼70% coverage (Fig. 3E and Supplementary data). These results suggest that DNA-bound XPC-RAD23B does not significantly facilitate DNA binding by UV-DDB. We next tested the second possibility using an IP assay with purified proteins. UV-DDB was precipitated by 3xFLAG-XPC-RAD23B, indicating that UV-DDB can form a complex with XPC-RAD23B (Fig. 3F). To rule out the possibility of DNA contamination during protein purification, we treated the sample with DNase I, and this did not change the results (Supplementary Fig. S3C). Additionally, to examine the complex formation between UV-DDB and XPC-RAD23B, SPR assay was performed. UV-DDB was immobilized on the surface of the SPR sensor chip. Varying concentrations of XPC-RAD23B were injected. As XPC-RAD23B was introduced, the RUs increased in a concentration-dependent manner (Fig. 3G). Notably, during the dissociation phase, in which unbound XPC-RAD23B was washed away, the RUs signal remained largely stable, indicating minimal dissociation. This suggested that XPC-RAD23B forms a complex with UV-DDB even in the absence of DNA. These IP and SPR results were consistent with the previous data that UV-DDB forms a complex with XPC-RAD23B in solution [22]. Given that the binding affinity of XPC-RAD2B for undamaged DNA is higher than that of UV-DDB (Fig. 1B), we propose that XPC-RAD23B enhances the binding of UV-DDB to undamaged DNA when the two proteins are in a complex. Conversely, UV-DDB in this complex did not appear to promote the binding of XPC-RAD23B to undamaged DNA.

### UV-DDB can diffuse along DNA

**Figure 4.**
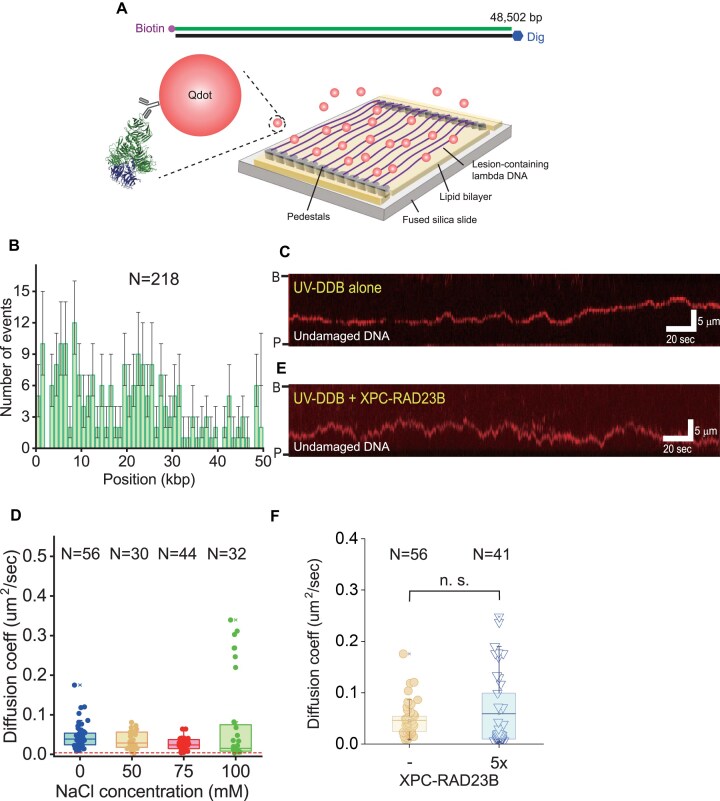
1D diffusion of UV-DDB on undamaged DNA. (**A**) Schematic of the double-tethered DNA curtain assay with undamaged λ-DNA. Biotinylated end of λ-DNA is anchored on lipid bilayer and stuck at the narrow barrier. The other end is modified with digoxigenin, which is tethered to anti-digoxigenin on the pedestals. (**B**) Initial binding position distribution of UV-DDB on undamaged DNA. The error bars are obtained from bootstrapping with 70% confidence interval. *N* indicates the total number of molecules analyzed. (**C**) Representative kymograph showing 1D diffusion of Qdot-labeled UV-DDB on undamaged DNA. B and P at the left indicate barrier and pedestal positions, respectively. (**D**) Diffusion coefficients as a function of NaCl concentration. *N* denotes the number of molecules analyzed. The red dashed line represents the theoretical upper limit of rotational diffusion coefficient around DNA helix. (**E**) Representative kymograph showing 1D diffusion of Qdot-labeled UV-DDB along DNA in the presence of unlabeled 5× XPC-RAD23B on undamaged DNA at 0 mM NaCl. B and P at the left indicate barrier and pedestal positions, respectively. (**F**) Diffusion coefficients of UV-DDB in the presence of XPC-RAD23B at 0 mM NaCl. 5× denotes five times higher molar concentration of XPC-RAD23B than that of UV-DDB. *N* denotes the number of molecules analyzed. The diffusion coefficients between the presence and the absence of 5× XPC-RAD23B are not statistically different (n.s.). The *P*-value of Student's *t*-test is 0.24.

We showed that the UX-complex facilitates UV-DDB binding to undamaged DNA and CPD-containing DNA (Fig. 1F–H). Given that XPC-RAD23B has poor ability to distinguish CPD from duplex DNA (Fig. 1B), the increased association of UV-DDB to undamaged DNA does not fully explain the enhancement of CPD recognition of UV-DDB by XPC-RAD23B. Hence, we compared the damage search mechanism of UV-DDB and the UX-complex using double-tethered DNA curtain assays, in which both ends of DNA were anchored at nanostructures. In this setup, DNA molecules remained stretched without buffer flow, allowing for the observation of the behavior of UV-DDB and/or XPC-RAD23B on DNA without interference from hydrodynamic force (Fig. 4A) [33]. For undamaged DNA, we observed a random distribution of UV-DDB on DNA, indicating that the initial binding of UV-DDB to DNA is sequence-independent (Fig. 4B). We found that UV-DDB moved along DNA through one-dimensional (1D) diffusion (Fig. 4C and Supplementary data). Based on single-molecule particle tracking, the relative displacement fits well to a single Gaussian function with the center at zero, proving that UV-DDB follows Brownian motion (Supplementary Fig. S4A–C). Estimated from mean square displacement, the mean 1D diffusion coefficient was ∼0.025 μm^2^/s, which was smaller than that of UV-DDB (K244E) reported previously (Supplementary Fig. S4D) [24]. The diffusion coefficients were insensitive to ionic strength, suggesting that UV-DDB diffuses along DNA not via hopping but via sliding (Fig. 4D) [25, 34]. Furthermore, the theoretical upper limit of diffusion coefficient for rotational motion around DNA helix was 0.003 μm^2^/s, which was smaller than experimental diffusion coefficients, implying that UV-DDB translationally slides along DNA without helical rotation (Fig. 4D and Supplementary data) [34, 35].

We previously reported that XPC-RAD23B diffuses along DNA via hopping [25]. This raised the question of how the UX-complex formation influences the diffusion behavior of UV-DDB. The initial binding position of UV-DDB in the presence of XPC-RAD23B with unlabeled XPC-RAD23B at a five times higher molar concentration (5×) was sequence-independent because both UV-DDB and XPC-RAD23B initially bind random sequences (Supplementary Fig. S4E). We then examined the diffusion coefficient of UV-DDB in the presence of XPC-RAD23B. We first attempted a two-color experiment in which UV-DDB and XPC-RAD23B were labeled with orthogonal Qdots but failed to form the complex possibly due to the large size of the Qdots. Therefore, we conducted the experiment with only UV-DDB in Q-dot labeled form (Fig. 4E and Supplementary Fig. S4F). In the presence of 5× XPC-RAD23B, the diffusion coefficient of UV-DDB was not significantly altered at 0 or 50 mM NaCl (Fig. 4F and Supplementary Figs S4G). This suggested that the diffusion coefficient of the UX-complex depends on UV-DDB or that UV-DDB diffuses alone without XPC-RAD23B.

### UV-DDB searches for CPD via both direct binding and 1D diffusion

**Figure 5.**
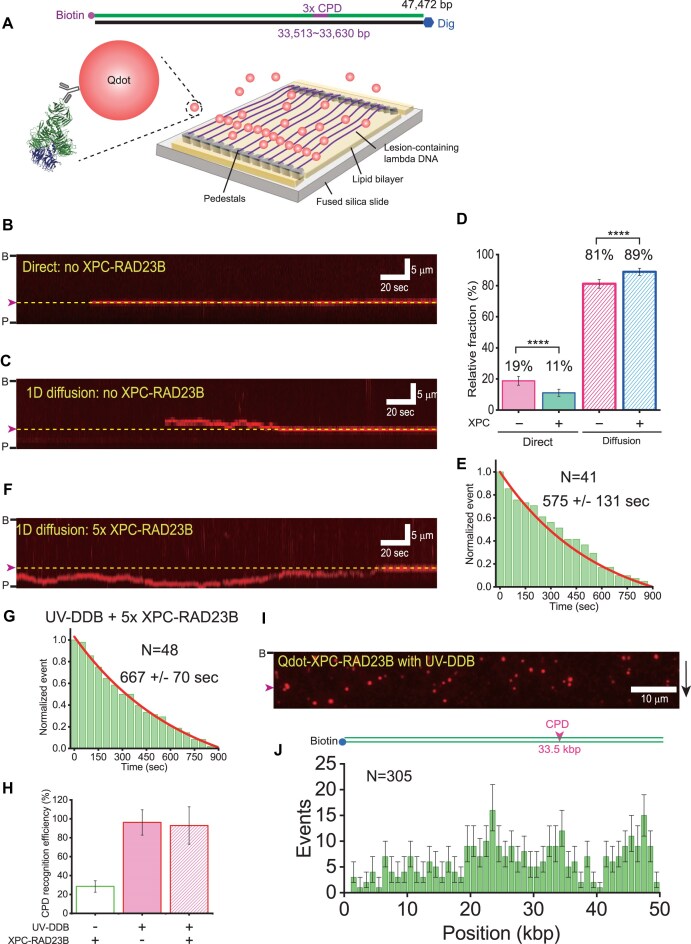
Damage search mechanism of UV-DDB. (**A**) Schematic of the double-tethered DNA curtain assay with λ-DNA construct containing triple CPD sites (3× CPD), which locates between 33 513 and 33 630 bp. (**B**) Representative kymograph showing direct binding (3D collision) of 50 pM UV-DDB to CPD in the absence of 250 pM XPC-RAD23B. Magenta arrowhead and yellow dashed line indicate the location of 3× CPD. (**C**) Representative kymograph showing CPD recognition of 50 pM UV-DDB through 1D diffusion in the absence of 250 pM XPC-RAD23B. Magenta arrowhead and yellow dashed line indicate the location of 3× CPD. (**D**) Relative population of direct binding to CPD (direct) and 1D diffusion (diffusion) of Qdot-UV-DDB depending on 5× XPC-RAD23B. The standard deviation is obtained from binomial probability distribution. The number of molecules analyzed is 133 and 144 in the presence and absence of 5× XPC-RAD23B, respectively. **** represents *P*-value <.0001 from Student's *t*-test. (**E**) Residence time analysis of UV-DDB at 3× CPD site. The normalized distribution is fitted by a single exponential decay function with 575 ± 131 s residence time, which is underestimated. *N* denotes the total number of molecules analyzed. (**F**) Representative kymograph showing CPD recognition of 50 pM UV-DDB through 1D diffusion in the presence of 250 pM XPC-RAD23B. Magenta arrowhead and yellow dashed line indicate the location of 3*×* CPD. (**G**) Residence time analysis of UV-DDB at 3× CPD site in the presence of XPC-RAD23B. The normalized distribution is fitted by a single exponential decay function with 667 ± 70 s residence time, which is underestimated. (**H**) CPD recognition efficiency of XPC-RAD23B alone and UV-DDB with and without 5× XPC-RAD23B through 1D diffusion. The efficiency is estimated as $\sum \frac{1}{{{\mathrm{bypass\ number}} + 1}} \times \frac{{{\mathrm{number\ of\ molecules\ for\ the\ bypass}}}}{{{\mathrm{total\ number\ of\ molecules}}}} \times 100{\mathrm{\ }}( {\mathrm{\% }} )$ (bypass number ≥0). The recognition efficiency of XPC-RAD23B alone was adopted from the previous study [25]. (**I**) Fluorescence images of 0.1 nM Qdot-labeled XPC-RAD23B with 0.1 nM unlabeled UV-DDB in the single-tethered DNA curtains under the buffer flow. The black bar and magenta arrowhead at the left represent the barrier and CPD positions, respectively. The black arrow at the right indicates the flow direction. (**J**) (Top) λ-DNA construct containing CPD sites around 33.5 kbp. (Bottom) Binding position distribution of 0.1 nM Qdot-labeled XPC-RAD23B in the presence of 0.1 nM unlabeled UV-DDB. Error bars are obtained from bootstrapping with 70% confidence interval. *N* indicates the total number of analyzed molecules.

We next examined how UV-DDB finds CPD using 3× CPD-containing λ-DNA (Fig. 5A and Supplementary Fig S2B and C). Immediately after injecting Qdot-labeled UV-DDB into a DNA curtain chamber, we stopped the flow and observed the behavior of UV-DDB on DNA. We found that UV-DDB either bound to 3× CPD directly or recognized the lesions via 1D diffusion (Fig. 5B and C). Nineteen percent of UV-DDB molecules directly associated with CPD while 81% of molecules showed 1D diffusion (Fig. 5D). We should note that the spatial resolution of our DNA curtain system was ∼1 kbp/pixel. Hence, the possibility that UV-DDB diffused within 1 kbp to find the lesion could not be excluded, and this might lead to an underestimation in the diffusion fraction. After binding to the CPD, UV-DDB remained stably associated with the lesion until the end of measurement. We estimated the residence time of UV-DDB at the CPD lesion to be 575 ± 131 s, an underestimation because some UV-DDB molecules remained on DNA until the end of our measurement (Fig. 5E). Considering the total time window (600 s), the residence time was indicative of stable binding of UV-DDB to CPDs.

We next examined how XPC-RAD23B modulates the damage search by UV-DDB (Fig. 5F). When XPC-RAD23B was co-injected in a five-fold molar excess concentration, direct binding events were reduced by about half to 11% and a concurrent increase in diffusion events was observed (Fig. 5D). This supported our suggestion that the UX-complex promotes the binding to DNA and enhances the lesion search through 1D diffusion. However, the addition of 5× XPC-RAD23B did not significantly change the residence time (667 ± 70 s) of UV-DDB at the lesion, as the protein remained stably associated with CPDs (Fig. 5G). Moreover, we estimated CPD recognition efficiency of UV-DDB from the kymographs by counting how often UV-DDB bypassed the CPD site before stable association (Fig. 5H). For 3× CPD, UV-DDB showed very high recognition efficiency (94%). Compared with the CPD recognition efficiency of XPC-RAD23B from our previous study, the recognition efficiency was three times higher [25]. Our data are consistent with the previous reports that UV-DDB specifically recognizes CPDs [14, 15]. The observation that the presence of XPC-RAD23B did not change the CPD recognition efficiency of UV-DDB is consistent with our model that XPC-RAD23B changes the search for, but not the binding of UV-DDB to lesions.

We also examined the binding of XPC-RAD23B to CPD in the presence of UV-DDB using DNA curtains. XPC-RAD23B was labeled with anti-FLAG-conjugated Qdot and incubated with DNA curtains along with unlabeled UV-DDB. No preferential binding of XPC-RAD23B to CPD was observed in the presence of UV-DDB (Fig. 5I and J), consistent with our EMSA data (Fig. 2D and E). The results are consistent with earlier findings that the transfer of CPDs from UV-DDB to XPC-RAD23B is a complex process that is regulated by ubiquitination [22].

### XPC-RAD23B reduces the intracellular mobility of UV-DDB

**Figure 6.**
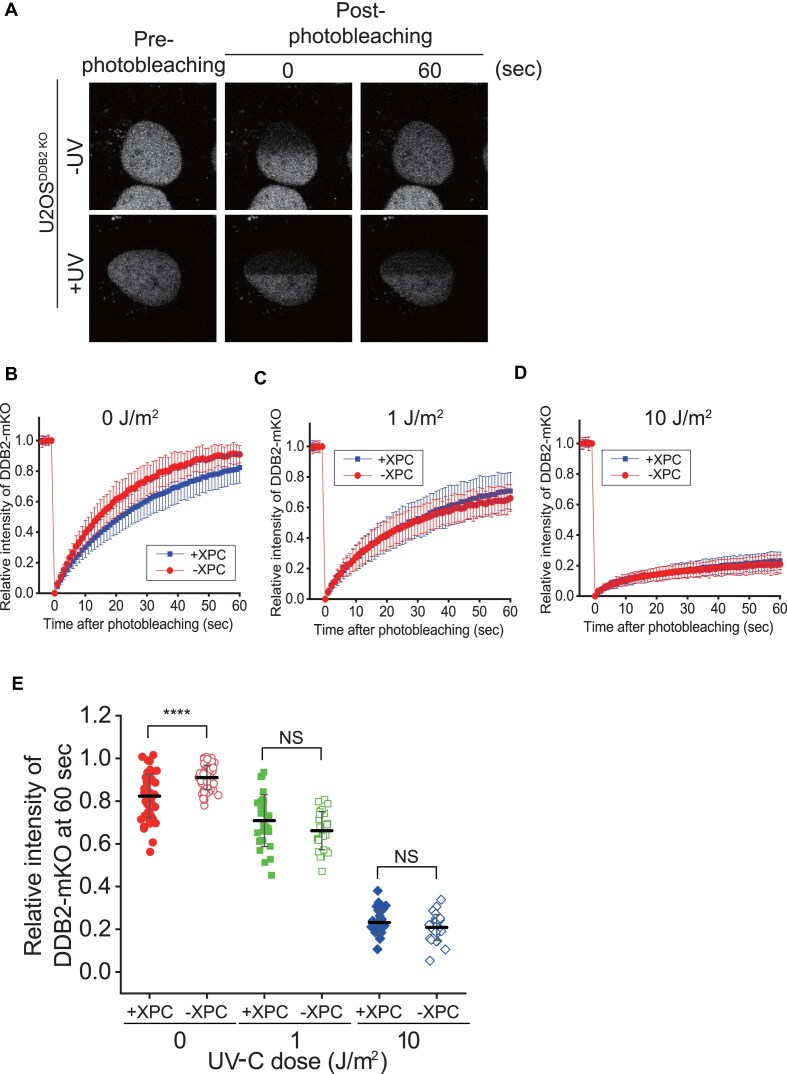
Intracellular behavior of UV-DDB and XPC-RAD23B. (**A**) FRAP of DDB2-mKO1 in live U2OS cells, where endogenous DDB2 is knocked out. The cells are mock-irradiated (−UV) or irradiated by 10 J/m^2^ of UV-C (+UV). Quantified FRAP signal of DDB2-mKO1 as a function of time in the presence (DDB2 KO) or absence (DDB2/XPC DKO) of XPC depending on different doses of UV irradiation [(**B**): 0 J/m^2^, (**C**): 1 J/m^2^, and (**D**): 10 J/m^2^].The fluorescence intensity is normalized by the fluorescence intensity of the pre-photobleaching state. (**E**) Relative fluorescence recovery at 120 s after photobleaching. ****: *P*-value ≤.0001 in Student's *t*-test. +XPC: DDB2 KO; −XPC: DDB2/XPC DKO.

We then tested whether the cooperative interaction between UV-DDB and XPC-RAD23B can also be observed in an intracellular environment. To this end, we knocked out the DDB2 gene and expressed mKO1-labeled DDB2 (DDB2-mKO1) in XPC-containing or XPC-depleted U2OS cells. We chose the cells that expressed DDB2-mKO1 at the same level as endogenous DDB2 in wild-type cells (Supplementary Fig. S5). We conducted FRAP for DDB2-mKO1 in the presence or absence of UV-C (Fig. 6A) [29, 36]. In the absence of UV-C irradiation, the mobility of DDB2-mKO1 without XPC [DDB2/XPC double-knockout (DKO)] was higher than that in the presence of XPC [DDB2 knockout (KO)], consistent with a complex formation of UV-DDB and XPC (Fig. 6B and E). When the cells were exposed to UV-C (1 and 10 J/m^2^), the mobility of DDB2-mKO1 was reduced and did not change with the presence of XPC, indicating that XPC does not affect the mobility of DDB2 following UV irradiation (Fig. 6C–E). External UV irradiation generates various UV lesions, including CPDs and 6-4PPs. As the dose of UV-C increased, the mobility of DDB2 decreased, suggesting that DDB2 molecules are rapidly engaged with UV lesions [37]. Figure 3C showed that the enhancement was mitigated when UV-DDB already bound to CPD prior to the UX-complex. Therefore, upon UV-C, the DDB2 mobility would not be influenced by XPC presumably because UV-DDB molecules already bind to UV lesions. However, we cannot exclude the possibility that other factors are responsible for the reduction of DDB2’s mobility independently of XPC. By contrast, the mobility of XPC in cells is already lower than that of other NER factors such as TFIIH, suggesting that XPC transiently associates chromatin and does not freely diffuse within the nucleus [38]. This could explain why the presence of DDB2 hardly affected the XPC mobility in previous FRAP experiments [39].

## Discussion

### XPC-RAD23B enhances the binding of UV-DDB to DNA by forming the complex

**Figure 7.**
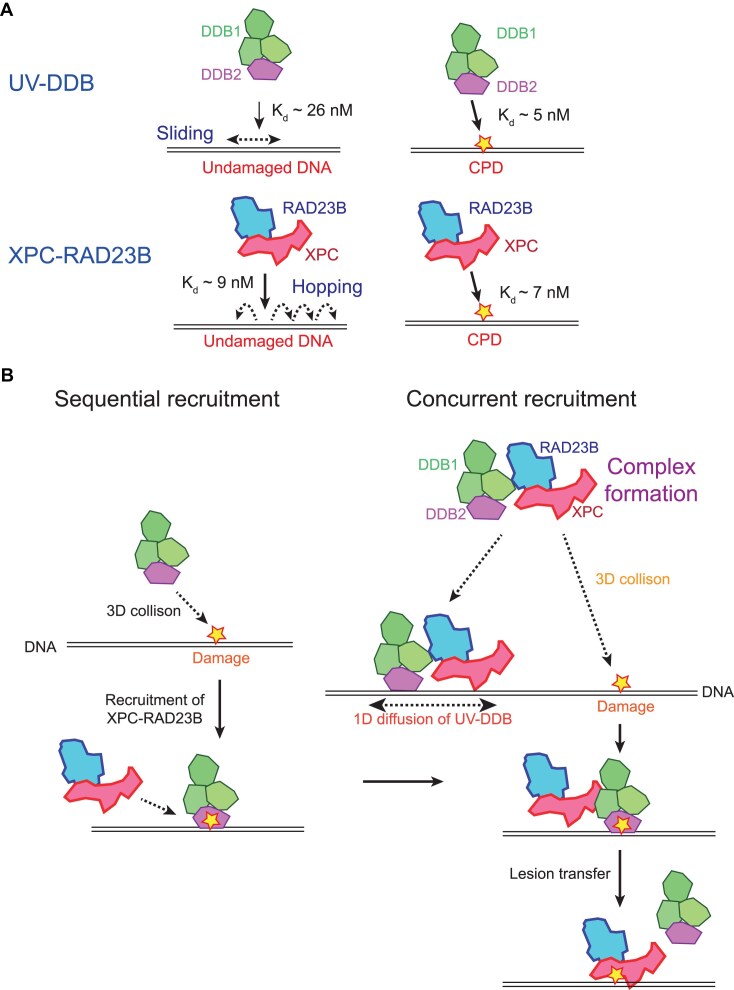
Model of damage sensing by UV-DDB and XPC-RAD23B. (**A**) Different binding affinity of UV-DDB and XPC-RAD23B to CPD and undamaged DNA, respectively. UV-DDB preferentially binds to CPD with higher binding affinity (*K*_d_ ∼ 5 nM) than to undamaged DNA (*K*_d_ ∼ 26 nM). On the other hand, XPC-RAD23B has similar binding affinity to either CPD (*K*_d_ ∼ 7 nM) or undamaged DNA (*K*_d_ ∼ 9 nM) and cannot distinguish CPD from DNA duplex. (**B**) Damage search mechanism by the complex of UV-DDB and XPC-RAD23B (UX-complex). In the current sequential recruitment model, UV-DDB recognizes CPDs first and then recruits XPC-RAD23B (left). In our new model, which is named as “concurrent recruitment” model, UV-DDB and XPC-RAD23B form the complex (UX-complex). The UX-complex can directly bind to CPD because UV-DDB also directly binds to CPD. In addition, XPC-RAD23B in the UX-complex has high binding affinity to undamaged DNA, promoting the binding of UV-DDB to DNA. Then UV-DDB alone or the UX-complex diffuses along DNA. The increased loading of UV-DDB onto DNA and the faster diffusion of the UX-complex or UV-DDB alone facilitate the search for CPD. After UV-DDB and XPC-RAD23B simultaneously bind to CPD, the lesion is transferred from UV-DDB to XPC-RAD23B.

XPC-RAD23B has a low ability to distinguish CPD-containing DNA from undamaged DNA, and hence UV-DDB is indispensable for the efficient repair of CPDs [16]. It is known that UV-DDB senses CPDs in chromatin and transfers the UV lesion to XPC-RAD23B in ubiquitination-dependent process [22]. However, how the two proteins cooperatively interact for lesion search has remained elusive. Combining single-molecule imaging and biochemical assays, we discovered that XPC-RAD23B enhances the binding of UV-DDB to DNA, facilitating CPD search. Interestingly, we did not observe any stimulation of XPC-RAD23B’s binding to DNA by UV-DDB. Our studies attribute this enhancement to (i) complex formation between UV-DDB and XPC-RAD23B (UX-complex), and (ii) a difference in DNA binding affinity and lesion discrimination ability of the two proteins. Our *in vitro* IP and SPR showed that UV-DDB and XPC-RAD23B directly interact to form the UX-complex (Fig. 3F) in line with a previous report [22]. Moreover, we found that the UX-complex binds to CPDs *in vitro*, consistent with a previous report that both UV-DDB and XPC-RAD23B are recruited to UV damage sites marked with CPD inside a cell [10]. So how does the complex formation enhance the UV-DDB binding to DNA? Our biochemical assays showed that XPC-RAD23B has higher binding affinity for undamaged DNA than UV-DDB, whereas it does not discriminate between CPDs and regular duplex DNA. By contrast, UV-DDB preferentially binds to CPD, while its binding affinity to undamaged DNA is lower than that of XPC-RAD23B (Figs 1B and 7A). Therefore, by forming the UX-complex, XPC-RAD23B can help UV-DDB associate with DNA, whereas the UX-complex does not promote XPC-RAD23B binding to DNA. In addition, it must be noted that a sub-stoichiometric amount of XPC-RAD23B could enhance the binding of UV-DDB to DNA at high concentrations, implying that XPC-RAD23B repeatedly interacts with UV-DDB to increase the UV-DDB binding (Fig. 1C and E).

Our FRAP assay showed the possibility of UX-complex formation in cells in the absence of excessive external UV irradiation. A cell contains about 100 000 DDB2 molecules [37, 40]. 1 J/m^2^ UV-C exposure can generate ∼0.027 photolesion per kbp, which amounts to ∼82 000 photolesions for the entire human genome (∼3 × 10^9^ bp) [41]. Even at 1 J/m^2^ UV-C exposure, almost all DDB2 molecules might be bound to photolesions and immobilized. Hence, the DDB2 mobility is not affected by XPC under UV irradiation in our FRAP experiments (Fig. 6B and E). By contrast, environmental UV exposure can produce ∼100 000 DNA lesions per cell per day [42]. In a previous study, simulated solar light generated 100 times fewer UV lesions in DNA at the same dose of UV-C exposure [41]. Therefore, daily UV exposure only produces only small number of photolesions, and the UV-DDB mobility was reduced by XPC due to UX-complex formation in the absence of UV.

### UV-DDB diffuses along DNA for the lesion search

The binding enhancement of UV-DDB to DNA by XPC-RAD23B does not account for the enhanced CPD recognition. Therefore, we investigated how UV-DDB or the UX-complex finds the UV lesions in DNA. Using single-molecule DNA curtain assays, we discovered that wild-type UV-DDB searches for DNA lesions via 1D diffusion along DNA as well as direct engagement with CPD (Fig. 5B and C). The diffusion is translational sliding without rotation around DNA helix (Fig. 4D). Structurally, when UV-DDB recognizes CPD, the dinucleotide of CPD is flipped into UV-DDB, which is facilitated by conformational flexibility at the lesion as well as by increased mobility in direct proximity of the lesion [12, 43]. Therefore, to find DNA damage, 1D diffusion of UV-DDB seems to be sufficient for DNA damage recognition. On the other hand, a previous study using single-molecule tightrope assays reported that wild-type UV-DDB is engaged with UV damage as a dimer only through direct binding (3D collision) without 1D diffusion [24]. However, the K244E-mutant UV-DDB, which is deficient in specific damage recognition, displayed 1D diffusion. We currently do not have a satisfactory explanation for the difference between these and our results. We suspect that different experimental conditions such as Qdot labeling or ionic strength of buffer might cause the discrepancy. On the other hand, our diffusion coefficient of wild-type UV-DDB was ∼0.025 μm^2^/s, which was four times smaller than that of the K244E mutant. The smaller diffusion coefficient of wild-type UV-DDB suggests that the wild-type protein binds more tightly to DNA, and therefore a smaller chance to miss the lesion.

The presence of XPC-RAD23B did not change the diffusion coefficients of UV-DDB even though XPC-RAD23B has higher diffusion coefficients (>0.1 μm^2^/s) than UV-DDB (Fig. 4F) [25, 44]. These results suggested that UV-DDB moves independently along DNA or determines the diffusion when it forms the UX-complex with XPC-RAD23B. Given the fact that XPC-RAD23B diffuses via hopping, the diffusion of the UX-complex follows UV-DDB diffusion because UV-DDB maintains the contact with DNA during diffusion while XPC-RAD23B frequently dissociates from and re-associates with DNA.

### The UV-DDB and XPC-RAD23B complex cooperatively searches for lesions

In the current sequential recruitment models, UV-DDB recognizes CPDs and then recruits XPC-RAD23B (Fig. 7B). Our biochemical assays showed that UV-DDB and XPC-RAD23B can form a complex in solution and bind at CPD as the complex. In addition, single-molecule imaging demonstrated that wild-type UV-DDB diffuses along DNA. Based on our results, we propose a new “concurrent recruitment” model for the damage search mechanism of UV-DDB and/or XPC-RAD23B (Fig. 7B). Since UV-DDB preferentially binds to CPD, UV-DDB in the UX-complex can directly engage with CPD though 3D collision. Alternatively, XPC-RAD23B in the UX-complex may bind undamaged DNA first. UV-DDB and XPC-RAD23B either diffuse together as a complex or move separately. Subsequently, UV-DDB alone or the UX-complex can bind to CPD. This is supported by Fig. 2A, where the intensity of 2× UV-DDB and the UV-DDB-XPC-RAD23B bands in the EMSA increased with the increasing UV-DDB concentration. If UV-DDB or XPC-RAD23B cannot move separately, 2× UV-DDB band formation would not be enhanced. Our DNA curtain assays showed that CPD recognition efficiency of the UX-complex is similar to that of UV-DDB, suggesting that the CPD recognition is determined by UV-DDB and not by XPC-RAD23B. Therefore, the CPD preference of UX-complex is similar to that of UV-DDB. On the other hand, XPC-RAD23B did not exhibit any preferential binding to CPDs even in the presence of UV-DDB, suggesting that the UX-complex does not guarantee XPC-RAD23B binding to CPD or CPD transfer from UV-DDB to XPC-RAD23B (Fig. 5I and J). These results are consistent with the previous study that CPD handover from UV-DDB to XPC-RAD23B requires ubiquitination of both proteins [22].

Many previous studies have established the mechanism of DNA damage recognition and NER initiation [14, 22, 45, 46]. UV lesions such as CPD are first recognized by UV-DDB and then transferred to XPC-RAD23B. Our model provides another pathway for the lesion search of UV-DDB, in which it gets assistance from XPC-RAD23B for DNA binding and then diffuses along DNA for lesion search. Considering the affinity of UV-DDB for CPDs in chromatin, this pathway may be active in open chromatin structures.

## Supplementary Material

gkaf463_Supplemental_Files

## Data Availability

The data underlying this article are available in the article and in its online supplementary material.

## Supplementary data

Supplementary data is available at NAR online.
