# Supplementary material for: XPC-RAD23B enhances UV-DDB binding to DNA to facilitate lesion search in nucleotide excision repair
Source: Nucleic Acids Res. 2025 Jun 18;53(11):gkaf463. doi: 10.1093/nar/gkaf463 (PMC12203913; doi:10.1093/nar/gkaf463)
Supplement: gkaf463_Supplemental_Files [file gkaf463_supplemental_files.zip › SupplementaryInformation.docx]

–

**Supplementary Information**

**XPC-RAD23B enhances UV-DDB binding to DNA to facilitate lesion search in nucleotide excision repair**

Soyeong An^1^, Masayuki Kusakabe^2^, Hyun-Suk Kim^3^, Hidetsugu Kozono^2^, Na Young Cheon^1^, Jeongeun Kim^4,5^, Jieun Kang^4^, Sunbok Jang^4, 5^, Kaoru Sugasawa^2^, Orlando D Schärer^1, 3, 6, 7^, and Ja Yil Lee^1, 3^

^1^ Department of Biological Sciences, Ulsan National Institute of Science and Technology, Ulsan 44919, Republic of Korea

^2^ Biosignal Research Center and Graduate School of Science, Kobe University, Kobe, Hyogo 657-8501, Japan

^3^ Institute of Basic Science Center for Genomic Integrity, Ulsan, 44919, Republic of Korea

^4^ College of Pharmacy, Graduate School of Pharmaceutical Sciences, Ewha Womans University, Seoul 03760, Republic of Korea

^5^ Graduate Program in Innovative Biomaterials Convergence, Ewha Womans University, Seoul, 03760, Republic of Korea

^6^ Graduate School of Health Science and Technology, Ulsan National Institute of Science and Technology, Ulsan 44919, Republic of Korea

^7^ Department of Pharmacology and Chemical Biology & Hillman Cancer Center, University of Pittsburgh Medical School, Pittsburgh, PA 15213, USA

Corresponding author: Ja Yil Lee: [biojayil@unist.ac.kr](mailto:biojayil@unist.ac.kr)

**Key words**: nucleotide excision repair, UV-DDB, XPC-RAD23B, DNA curtain, and damage search mechanism

**Running title**: Cooperative interaction between UV-DDB and XPC-RAD23B for CPD lesion search

**Contents**

**I. Supplementary Materials and Methods**

***1. DNA preparation***

*1.1. EMSA DNA preparation*

*1.2. Lambda DNA preparation*

***2. Protein purification***

*2.1. UV-DDB*

*2.2. XPC-RAD23B*

***3. Single-molecule DNA curtain assay***

***4. DNA curtain data analyses***

*4.1. Quantification of number of DNA-bound UV-DDB*

*4.2. Initial binding distribution of UV-DDB*

*4.3. Calculation of diffusion coefficients and theoretical upper limit of diffusion coefficient for helical rotation*

***5. Biochemical assays***

*5-1. Electrophoretic mobility shift assay (EMSA) for UV-DDB or/and XPC-RAD23B*

*5-2. Native gel western blot assay*

*5-3. In vitro immunoprecipitation assay*

***6. Fluorescence recovery after photobleaching (FRAP) assay***

*6-1. Cell culture and stable protein expression*

*6-2. immunoblot assay*

*6-3. FRAP experiments*

**II. Supplementary Table.** Sequences for synthesized oligomers

**III. Supplementary Figures**

**1. Supplementary Figure S1. Purification of UV-DDB and XPC-RAD23B and activity test**

**2. Supplementary Figure S2. Single-tethered DNA curtain assay and preparation of CPD-containing lambda DNA**

**3. Supplementary Figure S3. Effect of UV-DDB on the XPC-RAD23B binding to CPD**

**4. Supplementary Figure S4. Behavior of UV-DDB on undamaged DNA**

**5. Supplementary Figure S5. Expression of DDB2-mKO1 in U2OS cells**

**IV. Supplementary References**

**V. Supplementary Movie.** Movie for diffusion of UV-DDB on a single DNA molecule

**I. Supplementary Materials and Methods**

***1. DNA preparation***

All oligomer information was listed in Supplementary Table.

*1-1. EMSA DNA preparation*

The 39-mer oligomer (λ-I3_Dup) was purchased from Bionics (South Korea) (Supplementary Table) and its complementary oligomers labeled with FAM (λ-I3_comp_FAM) and unlabeled (λ-I3_comp_dark) were synthesized from Bioneer (South Korea) (Supplementary Table). The oligomer containing a CPD (λ-I3_CPD) was synthesized by Gene Link (USA) (Supplementary Table). λ-I3_Dup or λ-I3_CPD was mixed with λ-I3_comp_FAM at 1.5:1 molar ratio. λ-I3_Dup was mixed with λ-I3_comp_dark at 1.5:1 molar ratio to make dark competitors. The mixture was heated up to 95°C and slowly cooled down to 23°C for annealing.

*1-2. Lambda DNA (*λ-DNA) *preparation*

For DNA curtain experiments, λ-DNA was prepared as described in the previous study (1). For undamaged DNA experiments, λ-DNA was purchased from New England Biolabs (NEB, N3011). For the single-tether DNA curtain experiments, Biotin COS-L (or Biotin COS-R) and COS-R (or COS-L) oligomers were mixed with λ-DNA at 100:1 molar ratio in 1x ligase buffer (Enzynomics, M001) (Supplementary Table). After heated at 65 °C for 20 min, the mixture was slowly cooled down and subsequently 20 unit/μl of T7 DNA ligase (Enzynomics, M001) was added for ligation. For the double-tethered DNA curtain experiments, Biotin COS-L (or Biotin COS-R) and Dig COS-R (or Dig COS-L) oligomers were ligated with the same protocol (Supplementary Table).

For lesion (CPD)-containing λ-DNA, λ-I3 (total 47,472 bp), which contains 7 nickase sites at a specific location (13,817 bp ~ 13,974 bp), was used, and the protocol was followed by the previous studies as depicted in Supplementary Figure S2A and S2B (1). Triple CPDs (3xCPD) were inserted into the 7 nickase sites in λ-I3. For that, 0.8 unit/μl of Nt.Bsp.QI nickase (NEB, R0644) was treated with 3 nM of λ-I3 at 50°C for 1 h to make a gap at a specific location on the λ-I3. The nickases were eliminated by 0.5 mg/ml of proteinase K, which was then heat-inactivated at 80°C for 20 min. Then, 1000-fold excessive CPD-containing oligomers (λ-I3_CPD, Gene Link), 300-fold excessive Biotin COS-R (or Biotin COS-L) and Dig COS-L (or Dig COS-R) were added and slowly cooled down to 23°C for annealing with the gapped λ-I3 and simultaneously ligated by 20 unit/μl of T4 DNA ligase. After heat inactivation at 65°C for 20 min, the unbound oligomers were filtered out with a gel filtration spin column (Illusta MicroSpin^TM^ S-400, GE Healthcare). The insertion was confirmed by NcoI digestion. The λ-I3 without CPDs was cut by NcoI, whereas the CPD-containing λ-I3 was not (Supplementary Figure S2B and S2C).

***2. Protein purification***

All proteins were purified at 4°C.

*2-1. UV-DDB*

UV-DDB purification was followed by the previous protocol with slight modifications (2). Human *DDB1* and *DDB2* genes were inserted into pFastBac Dual plasmid vector. For purification and fluorescent labeling, 1xFLAG and 3xHA tags were fused into the amino-terminus of *DDB1* gene, respectively. PreScission protease cleavage sequence was placed between 1xFLAG and 3xHA. Bacmid DNA was produced using MAX Efficiency DH10Bac Competent Cells (Thermo Fisher Scientific, 10361012). The pFastBac Dual construct containing *DDB1* and *DDB2* genes was transformed into DH10Bac cells, and bacterial clones harboring the recombined bacmid were selected by blue/white colony screening. The purified bacmid was used to transfect Sf9 insect cells for producing P1 virus. For amplification, High-Five insect cells were infected with the P1 virus, and then the resulting P2 virus was used to infect 0.4 L culture of High-Five cells for over-expression of the protein. The infected cells were harvested and resuspended in 80 ml of lysis buffer (50 mM Tris-HCl [pH 8.0], 1 mM EDTA, 0.5 M NaCl, 0.25 mM TCEP, 10% glycerol, and Halt protease inhibitor cocktail (Thermo Fisher Scientific, 78438). The resuspended cells were lysed by sonication and clarified by centrifugation at 148,000 g for 30 min. The clarified lysates were incubated with 2 ml of anti-FLAG M2 agarose beads (Merck, A2220) overnight. The beads were collected by centrifugation at 2,500 g for 5 min and washed with 20 ml of buffer PET (20 mM Na-phosphate [pH 7.8], 10 % glycerol, 1 mM EDTA, 0.01% Triton X-100, 1 mM dithiothreitol (DTT), and 0.25 mM PMSF) supplemented with 0.3 M NaCl. This washing step was repeated twice more. Then the beads were resuspended in 10 ml of buffer PET with 0.3 M NaCl and packed into a disposable column (Thermo Scientific, 29920) by gravity flow. The packed beads were washed with 5 ml of buffer PET supplemented with 0.1 M NaCl. UV-DDB was eluted 10 times with 1 ml fraction of 0.1 mg/ml of 1xFLAG peptide (Merck, F3290) in 0.1 M NaCl/buffer PET. The eluates were treated with 8.5 μg/ml of PreScission protease overnight to remove 1xFLAG from the amino terminus. The proteins were subsequently injected into a HiTrap heparin HP column (1 ml, Cytiva, 17040601) equilibrated in buffer PET containing 0.1 M NaCl. In this step, the DDB1-DDB2 complex was retained in the heparin column, while DDB1 free from DDB2 flowed through it. After the column was washed with 10 ml of buffer PET containing 0.1 M NaCl, bound UV-DDB was eluted with buffer PET containing 1 M NaCl. To reduce a salt concentration, the eluate from the heparin column was then flowed through a HiTrap desalting column (5 ml, Cytiva, 17140801) equilibrated with buffer PET containing 0.1 M NaCl, and loaded into a Mono Q 5/50 GL column (Cytiva, 29275878) equilibrated in buffer PET containing 0.1 M NaCl. During elution with a NaCl gradient from 0.1 M to 0.6 M in buffer PET, UV-DDB with 3xHA at the amino-terminus of DDB1 was eluted around 0.3 M NaCl. Then the purified proteins were stored at -80ºC until use.

The activity of purified UV-DDB was tested by a pull-down assay with UV-damaged DNA. For this purpose, pBluescript II KS (+) plasmid DNA (10 µg) was digested with NotI, and the ends were biotinylated by filling with T4 DNA polymerase (Takara Bio) in the presence of 100 µM each of dATP, dGTP, and dTTP, and 20 µM biotin-14-dCTP (Thermo Fisher Scientific, 19518018). Free nucleotides were removed by passing through a CentriSep spin column (Princeton Separations) equilibrated with TE buffer containing 0.1 M NaCl and 0.1% SDS. After ethanol precipitation, the biotinylated DNA was redissolved in TE buffer and an aliquot was exposed to UVC at 1 kJ/m^2^ under an array of germicidal lamps (Toshiba, GL-15, with a 254 nm peak). The UV-irradiated DNA and unirradiated control DNA were separately conjugated to streptavidin-coated paramagnetic beads (Dynabead^TM^ M-280 Streptavidin, Invitrogen, 11205D) using the Dynabeads kilobaseBINDER Kit (Thermo Fisher Scientific, 60101). The DNA-conjugated magnetic beads were suspended in buffer PET containing 0.1 M NaCl and incubated with the purified UV-DDB on ice for 1 hour. After the unbound fraction was recovered, the DNA-beads were washed extensively with the same buffer and then denatured by boiling with 1xSDS loading dye. The bound and unbound fractions of UV-DDB for undamaged and UV-damaged DNA were compared by western blot with anti-HA antibody.

*2-2. XPC-RAD23B*

We followed the previous purification protocol (1). Briefly described, XPC-RAD23B with 3xFLAG at amino-terminus of XPC was overexpressed in 0.4 L of insect cells (Sf9). After harvested, the cells were resuspended in 40 mL of Lysis buffer (10 mM PBS [pH 7.4], 500 mM NaCl, 1 mM PMSF, 0.3% Nonidet P-40 (NP-40), cOmplete™ EDTA-free Protease Inhibitor Cocktail (Roche, 5056489001), and 1 mM 2-mercaptoethanol (2ME)). The resuspended cells were lysed by Dounce homogenizer and then clarified by centrifugation (at 40,000 g for 30 min). The clarified lysates were incubated with anti-FLAG M2 agarose bead for 3.5 hours. The beads were collected by centrifugation at 2,500 g and washed twice with 10 ml, and then 4 times with 2 ml of washing buffer (10 mM PBS [pH 7.4], 500 mM NaCl, and 0.1% NP-40). Then the beads were packed in a gravity flow column, and XPC-RAD23B was eluted 6 times with one column volume (CV) of 0.2 mg/ml of 3xFLAG peptide (Merck, F4799) in Lysis buffer. The eluted proteins were subsequently purified through a gel filtration column (HiLoad16/600 Superdex 200, Pharmacia) equilibrated in GF buffer (25 mM potassium phosphate [pH 7.6], 10% glycerol, and 5 mM 2ME) supplemented with 200 mM NaCl. The eluted XPC-RAD23B was further purified and concentrated by heparin column (1 mL of HiTrap Heparin, Pharmacia). The proteins were eluted by a 10 ml linear NaCl gradient from 0.2 M to 1.5 M in GF buffer. The eluates were then dialyzed overnight against storage buffer (25 mM potassium phosphate [pH 7.6], 100 mM NaCl, 10% glycerol, and 5 mM 2ME) and then stored at -80°C after snap-freezing in liquid nitrogen.

The activity of purified XPC-RAD23B was tested by *in vitro* NER assay (1,3). 5 nM of XPC-RAD23B, 10 nM of TFIIH, 20 nM of XPA, 41.6 nM of RPA, 27 nM of XPG, and 13.3 nM of XPF-ERCC1 were incubated with a plasmid with a 1,3-intrastrand cisplatin adduct (1,3). All proteins were >95% pure and produced as previously described: TFIIH (4) ; XPA (5), RPA (6); XPG (7); ERCC1-XPF (8). The reaction was performed in repair buffer containing 45 mM HEPES-KOH [pH 7.8], 5 mM MgCl_2_, 0.3 mM EDTA, 40 mM phosphocreatine (di-Tris salt, Sigma, P1937), 2 mM ATP, 1 mM DTT, 2.5 μg BSA, 0.5 μg creatine phosphokinase (Sigma), and NaCl (to a final concentration of 70 mM). After pre-incubation at 30°C for 10 min, 1 μl of the cisplatin-containing plasmid DNA (50 ng) was added to the mixture, which was further incubated at 30°C for 45 min and then placed on ice. To quantify the excised NER products (cisplatin-containing oligonucleotides), 0.5 μl of 1 μM 35-nt oligonucleotide with the complementary sequence (5’-GGG GGA AGA GTG CAC AGA AGA AGA CCT GGT CGA CC-3’) was added, and the samples were heated at 95°C for 5 min and then cooled to 23°C to allow annealing. Then 1 μl of a Sequenase [⍺-^32^P]-dCTP mixture (0.5 units of Sequenase and 2.5 μCi of [⍺-32P]-dCTP per reaction) was added and incubated at 37°C for 3 min. Subsequently, 1.2 μl of dNTP mixture (100 μM each of dATP, dTTP, dGTP, and dCTP plus 50 μM dCTP) was added and incubated at 37°C for 12 min to extend the excised product with radiolabeled dCTP. The reactions were stopped by adding 8 μl of loading dye (90% formamide and 10 mM EDTA) and heating at 95°C for 5 min. The samples were loaded on a 14% sequencing gel (0.5x TBE) and run at 45 W for 2.5 hours. The reaction products were visualized using a PhosphorImager (Typhoon RGB, GE Healthcare).

***3. Single-molecule DNA curtain assay***

The single-molecule DNA curtain assay was conducted as described before (9,10). All DNA curtain experiments were performed at 23°C. Prism-type total internal reflection fluorescence microscope (TIRFM) was custom-built with Nikon Eclipse Ti-2. The solid-state 488-nm (200 mW, OBIS, Coherent Laser) was used to excite Qdots and Alexa488-labeled antibodies. Fluorescence signal was split by a dichroic mirror and imaged into two electron-multiplying CCD cameras (iXon 897, Andor Technology). The data were collected by the NIS-Element software (Nikon).

For the single-tethered DNA curtain assay, a nano-trench patterned slide was used for a flowcell (11). λ-DNA modified with only biotin at one end was anchored on the biotinylated lipid bilayer via streptavidin. DNA curtains were formed at nano-trench patterns under hydrodynamic force by continuous buffer flow at 0.3~0.5 ml/min of flow rate (Figure 1E).

To fluorescently label purified UV-DDB, HA-antibody (Invitrogen, 26183) conjugated quantum dots (Qdots) were prepared using 705 nm Qdot Antibody Conjugation Kit (SiteClick Qdot Antibody Labeling Kits, Thermo Fisher Scientific, S10454). 3xHA-tagged UV-DDB was incubated with HA-antibody conjugated Qdots at 1:3 molar ratio on ice for at least 10 min. Qdot-labeled UV-DDB was diluted in imaging buffer (10 mM Tris-HCl [pH 8.0], 1 mM EDTA, 0.16% BSA, 1 mM DTT with 0 ~ 100 mM NaCl). The injection was stopped when a maximum number of proteins reached the DNA curtains, and proteins were incubated in the flowcell for 10 min at 23°C. To test the enhancement of UV-DDB binding to DNA by XPC-RAD23B, 0.2 nM of Qdot-labeled UV-DDB and 0.4 nM XPC-RAD23B were injected together and incubated in the flowcell. After the flow was resumed, UV-DDB binding was observed. The protein binding was ensured by transient switch-off’s, at which DNA was recoiled out of the evanescent field and bound proteins disappeared to be distinguished from surface-adsorbed molecules. To examine whether DNA-bound XPC-RAD23B recruited UV-DDB, 8 nM XPC-RAD23B was bound to DNA molecules in DNA curtains. Subsequently, XPC-RAD23B was labeled with 100 nM Alexa488-conjugated FLAG-antibodies (Invitrogen, MA1-142-A488). The coverage of XPC-RAD23B was estimated from EMSA data in Figure 1B. Structurally, single XPC-RAD23B occupies 30 bp of DNA (12). The EMSA for XPC-RAD23B with 4 nM of 39 bp undamaged DNA showed that approximately 70% of XPC-RAD23B was bound to DNA at 12.5 nM, suggesting that XPC-RAD23B has 70% coverage on DNA when the molar ratio between XPC-RAD23B and DNA is 2.5:1 for 39 bp DNA. We used 2 pM of lambda DNA with 48,502 bp for DNA curtains, which is equivalent to ~2.5 nM for 39 bp DNA. Therefore, the coverage of 8 nM of XPC-RAD23B was greater than 70%. After free XPC-RAD23B and Alexa488-FLAG antibodies were washed out, 0.2 nM Qdot-labeled UV-DDB was added to the XPC-RAD23B-covered DNA curtains. To test binding of XPC-RAD23B to CPD in the presence of UV-DDB using the DNA curtains, 3xFLAG-tagged XPC-RAD23B was labeled with anti-FLAG-conjugated Qdots, which was prepared in the same manner as anti-HA Qdots above using FLAG-antibody (Merck, F3165). Qdot-labeled XPC-RAD23B was mixed with equimolar unlabeled UV-DDB, and the mixture was diluted up to 0.1 nM in imaging buffer (10 mM Tris-HCl [pH 8.0], 1 mM EDTA, 0.16% BSA, and 1 mM DTT). The injection was stopped when a maximum number of proteins reached DNA curtains. Both proteins were then incubated in the flowcell at 23°C for 10 min. Qdot-labeled XPC-RAD23B was imaged after the flow was resumed.

For the double-tethered DNA curtain assay, a chromium-barrier slide, which has a diffusion barrier and pentagon-shaped pedestals, was used. λ-DNA modified with biotin at one end and digoxigenin at the other end was anchored on the biotinylated lipid bilayer via streptavidin. The digoxigenin-labeled end was tethered to the pedestal, on which anti-digoxigenin was adsorbed. Immediately after Qdot-labeled UV-DDB was injected into the DNA curtains, the flow was switched off and images were taken.

All images were taken at 10 frame per second rate. The images were converted into TIFF format and analyzed by Image J (NIH).

***4. DNA curtain data analyses***

All DNA curtain movies taken by NIS-Elements (Nikon) were converted to 16-bit TIFF stacks and analyzed in Image J.

*4.1. Quantification of number of DNA-bound UV-DDB*

To check salt effect about binding affinity of UV-DDB, we quantified the number of DNA-bound UV-DDB using single-tethered DNA curtains. First, we checked DNA molecules in DNA curtains by YOYO-1 staining. We built up kymographs of individual DNA molecules and counted the number of DNA molecules having fluorescence puncta, which disappeared when flow was turned off. We analyzed the number of DNA-bound UV-DDB more than three barriers. Each barrier of number of DNA-bound UV-DDB on each DNA molecule were averaged and we got the standard deviation of them to check error. The normalization of Figure 1F was carried out by dividing the average number of bound UV-DDB per DNA in the presence of XPC-RAD23B by that in the absence of XPC-RAD23B.

*4.2. Initial binding distribution of UV-DDB*

From a kymograph showing UV-DDB diffusion, the frame, at which UV-DDB began to bind to DNA, was considered as the initial binding frame. Then, at the initial binding frame, the fluorescence signal of Qdot was fitted by 2D Gaussian function in Image J and the vertical center position was considered as the initial binding position of UV-DDB on DNA. Then the binding distribution histogram was built up with 1 kbp bin size. The error bars were obtained from bootstrapping with 70% confidence interval.

*4.3. Calculation of diffusion coefficients and theoretical upper limit of diffusion coefficient for helical rotation*

The diffusion coefficients and theoretical upper limit of diffusion coefficient for helical rotation were calculated by the previous methods (1,13). Time traces of UV-DDB were obtained using MOSAIC suit particle tracker, an Image J plug-in (NIH). From the time traces, mean square displacement (*MSD*) was calculated using a home-built program of Matlab (Mathworks) based on the mathematical definition of MSD given as

$MSD \left( n, N \right)= \sum_{i=1}^{N-n} \frac{{(Y_{i+n}-Y_{i})}^{2}}{N-n}$,

where *N* is the total number of frames, *n* is the measurement window ranging from 1 to *N-1*, and *Y* is the position of UV-DDB along DNA. The standard deviation (*SD*) in *MSD*, which was considered as error, was given as

$SD=\sqrt{\frac{\left( 2Di\Delta t \right)^{2}(2t^{2}+1)}{3i(N-i+1)}}$.

The 1D diffusion coefficient (*D*) of each UV-DDB molecule was estimated from MSD based on the equation,

$MSD =2Dn\Delta t$.

Practically, the 1D diffusion coefficient *(D)* was obtained from the linear fitting of *MSD* with the first three data points because the error of *MSD* becomes large as the frame increases.

The theoretical upper limit of diffusion coefficient with helical rotation was calculated from the Einstein’s relation,

$D=\frac{k_{B}T}{\xi}$,

where *k_B_* is Boltzmann constant, *T* is absolute temperature, and ξ is the friction coefficient. For the helical rotation, ξ is modified like

$\xi=6\pi\eta R+ \left( \frac{2\pi}{10 BP} \right)^{2}\left[ 8\pi\eta R^{3}+6\pi\eta R\left( R_{OC} \right)^{2} \right]$,

where *η* is buffer viscosity, *R* is the radius of protein, *BP* is the distance between neighboring bases (0.34 nm for B-DNA), and *R_OC_* is the distance between DNA helical axis and the center of mass of the protein. In our DNA curtain experiments, UV-DDB was labeled with Qdot, and hence *R_OC_* is given as the sum of UV-DDB radius and the hydrodynamic radius of antibody-conjugated Qdot. The radius of UV-DDB is ~ 7 nm from its x-ray crystal structure (PDB ID: 4e5z) (14). The hydrodynamic radius of antibody-conjugated Qdot is ~ 13 nm based on the information of maker (Invitrogen). So, *R* and *R_OC_* are approximately 20 and 21 nm. *k_B_T* is approximated to 4.1 pN nm at 23°C. Finally, the rotational limit of diffusion coefficient is ~ 0.003 μm^2^/sec, which is by far smaller than the diffusion coefficients of UV-DDB at all measured NaCl concentrations, suggesting that UV-DDB translationally diffuses along the DNA backbone without rotation around the helix.

***5. Biochemical assays***

*5-1. Electrophoretic mobility shift assay (EMSA) for UV-DDB or/and XPC-RAD23B*

DNA was prepared as described in Section 1-1. To compare with binding affinity of UV-DDB and XPC-RAD23B at DNA damage or test binding affinity enhancement, we performed electrophoretic mobility shift assay (EMSA). All reactions were performed in reaction buffer (50 mM Tris-HCl [pH 7.5], 100 mM NaCl, 0.1 mg/ml BSA, and 1 mM DTT) at 23°C. For the binding affinity of either UV-DDB or XPC-RAD23B, 4 nM annealed DNA was incubated with each protein at different concentration in reaction buffer at 23°C for 45 min in a dark room to prevent photobleaching of FAM. For the EMSA with competitors, 200 nM unlabeled DNA oligomers (50x) were simultaneously added to the reactant. Except that, all other EMSA experiments were conducted with 10 nM DNA under the same condition. For the sequential incubation, 2 nM of XPC-RAD23B was pre-incubated with 10 nM DNA for 25 min and then UV-DDB was titrated with further 25-min incubation. In addition, UV-DDB at different concentrations was pre-incubated with 10 nM DNA and then 2 nM XPC-RAD23B was incubated for further 25 min. The reactions were analyzed by 6% non-denaturing polyacrylamide gel electrophoresis (PAGE), which was run at 20 mA using 0.5x TBE buffer at 4°C. The gel was imaged by Typhoon RGB (Cytiva). The band intensity was quantified by Gel Analysis tools in ImageJ (NIH). The bound fraction was calculated by dividing the sum of bound band intensities by the sum of bound and unbound band intensities.

*5-2. Native gel western blot*

To check XPC-RAD23B and UV-DDB complex, we performed native gel western blot. In here, we used same DNA and react in same condition. The reactions were loaded on 6% non-denaturing polyacrylamide gel electrophoresis (PAGE), which was run at 20 mA using 0.5x TBE buffer at 4°C. The gel was run for longer time (about 3 hours) to spread each band. The gel was imaged by Typhoon RGB to check band position on the gel. The shifted bands on the gel were transferred to the membrane to perform western blot. We used anti-HA antibody (1:10000) (Invitrogen, 26183) and XPC antibody (1:300) (SantaCruz, sc-74410) to test which bands are containing XPC-RAD23B or/and UV-DDB.

*5-3. In vitro immunoprecipitation*

To investigate direct interaction between UV-DDB and XPC-RAD23B, we tried *in vitro* immunoprecipitation using anti-FLAG M2 agarose beads. We pre-equilibrated 6 μl of anti-FLAG M2 agarose bead in NETN buffer (20 mM Tris-HCl [pH 8.0], 100 mM NaCl, 1 mM EDTA, 0.1% NP-40). 50 nM of XPC-RAD23B were incubated in 30 μl of total reaction volume with pre-equilibrated anti-FLAG M2 agarose beads and NETN buffer at 4°C overnight. The beads were collected by centrifugation at 7,700 g for 1 min and washed with 500 μl of NETN buffer. This washing step was repeated four times more. Then the XPC-RAD23B bound beads were incubated with 100 nM of UV-DDB in the presence or absence of 1 μg/ml DNase I (Merck, D5319-2) in 30 μl of total reaction volume with NETN buffer with 0.1 mg/ml BSA at 4°C for 1 hour. The beads were collected by mild centrifugation at 7,700 g for 1 min and washed with 500 μl of NETN buffer. This washing step was repeated four times more. The beads were incubated in 30 μl of NETN buffer with 0.1 mg/ml BSA containing 0.5 mg/ml 3xFLAG peptide (Peptron). The beads were collected by centrifugation at 7,700 g for 1 min and took off elutes. The elutes were checked by western blot with anti-XPC antibody (1:300) (SantaCruz, sc-74410) and anti-HA antibody (1:10000) (Thermo Fisher Scientific, 26183).

**6. Fluorescence recovery after photobleaching (FRAP) assay**

*6-1. Cell culture and stable protein expression*

Human osteosarcoma cell line U2OS was cultured in Dulbecco’s modified Eagle medium (DMEM) supplemented with 10% fetal bovine serum (FBS) at 37°C, 5% CO_2_ in humidified atmosphere. PiggyBac transposon vector system (System Biosciences) was used for the stable expression of DDB2-mKO1 in U2OS cells, in which endogenous DDB2 gene or both endogenous DDB2 and XPC genes were disrupted. The PB-CMV-MCS-EF1 Puro PiggyBac construct encoding DDB2-mKO1 and the Super PiggyBac transposase expression vector were co-transfected into U2OS cells with FuGene HD reagent (Promega). After culturing the cells for 3 days, stable transformants were selected in DMEM containing 1 μg/mL puromycin. Single clones were isolated by limiting dilution.

*6-2. Immunoblot assay*

For preparing cell extracts, cells were lysed for 60 min on ice with cytoskeleton (CSK) buffer (10 mM PIPES-NaOH [pH 6.8], 3 mM MgCl_2_, 1 mM EGTA, 0.3 M NaCl, 10% glycerol, and 0.1% Triton X-100) containing a protease inhibitor cocktail (1 μg/mL leupeptin, 2 μg/mL aprotinin, 1 μg/mL pepstatin, and 50 μg/mL Pefabloc SC, all purchased from Merck). Proteins were separated by SDS-PAGE and transferred onto polyvinylidene difluoride membranes. After blocking with 5% skimmed milk in TBS-T (50 mM Tris-HCl [pH 8.0], 150 mM NaCl, and 0.1% Tween 20), the membranes were incubated with primary antibodies diluted in the blocking solution. After washing with TBS-T, the membranes were incubated with the appropriate horseradish peroxidase-conjugated secondary antibody. Immunoreactive bands were visualized by chemiluminescence with the ImmunoStar Zeta (Fujifilm Wako Pure Chemical) as a substrate. The chemiluminescence signals were detected by the lumino-imaging analyzer LAS-3000 (Fujifilm).

*6-3. FRAP experiments*

Cells expressing DDB2-mKO1 were cultured in 35 mm glass bottom dishes (Mattek). Under the confocal laser microscope system (Evident, FV3000), a region of interest (ROI) covering approximately half area of nucleus was set for photobleaching. Before photobleaching, 4 images were acquired every 1 sec and then the fluorescence of mKO1 in the ROI was bleached with the 561 nm laser at 100% power. After photobleaching, the fluorescence images were acquired every 1 sec for 1 min, and data analyses were performed as described previously (15). For irradiating cells with UVC, the culture medium was removed, and the cells were washed with PBS and then irradiated with UVC at different doses (0, 1, or 10 J/m^2^). FRAP was performed within 30 min after UVC irradiation.

**II. Supplementary Table. List of oligomers and their sequences**

| **Name** | **Sequence** | **Manufacturer** |
| --- | --- | --- |
| COS-L | 5’-Phos-GGG CGG CGA CCT-3’ | Bioneer |
| COS-R | 5’-Phos- AGG TCG CCG CCC-3’ | Bioneer |
| Biotin COS-L | 5’-Phos-GGG CGG CGA CCT-biotin-3’ | Bioneer |
| Biotin COS-R | 5’-Phos- AGG TCG CCG CCC-biotin-3’ | Bioneer |
| Dig COS-L | 5’-Phos-GGG CGG CGA CCT-digoxigenin-3’ | Bioneer |
| Dig COS-R | 5’-Phos- AGG TCG CCG CCC-digoxigenin-3’ | Bioneer |
| λ-I3_Dup† | 5’-Phos-TGC ATG CGG CCG CTC TTC CCA TGG TGC GAT CGC TCT TCG -3’ | Bionics |
| λ-I3_CPD * | 5’-Phos-TGC ATG CGG CCG CTC TTC CC**T^T**GG TGC GAT CGC TCT TCG -3’ | Gene Link |
| λ-I3_comp_FAM | 5’-FAM-CGA AGA GCG ATC GCA CCA TGG GAA GAG CGG CCG CAT GCA-3’ | Bioneer |
| λ-I3_comp_dark | 5’-CGA AGA GCG ATC GCA CCA TGG GAA GAG CGG CCG CAT GCA-3’ | Bioneer |

† The sequence in green (CCA TGG) represents the NcoI cleavage site, which will be protected by the insertion of CPDs.

* The bold **T^T** represents CPD.

**III. Supplementary Figures**

**Supplementary Figure S1. Purification of UV-DDB and XPC-RAD23B and activity tests**


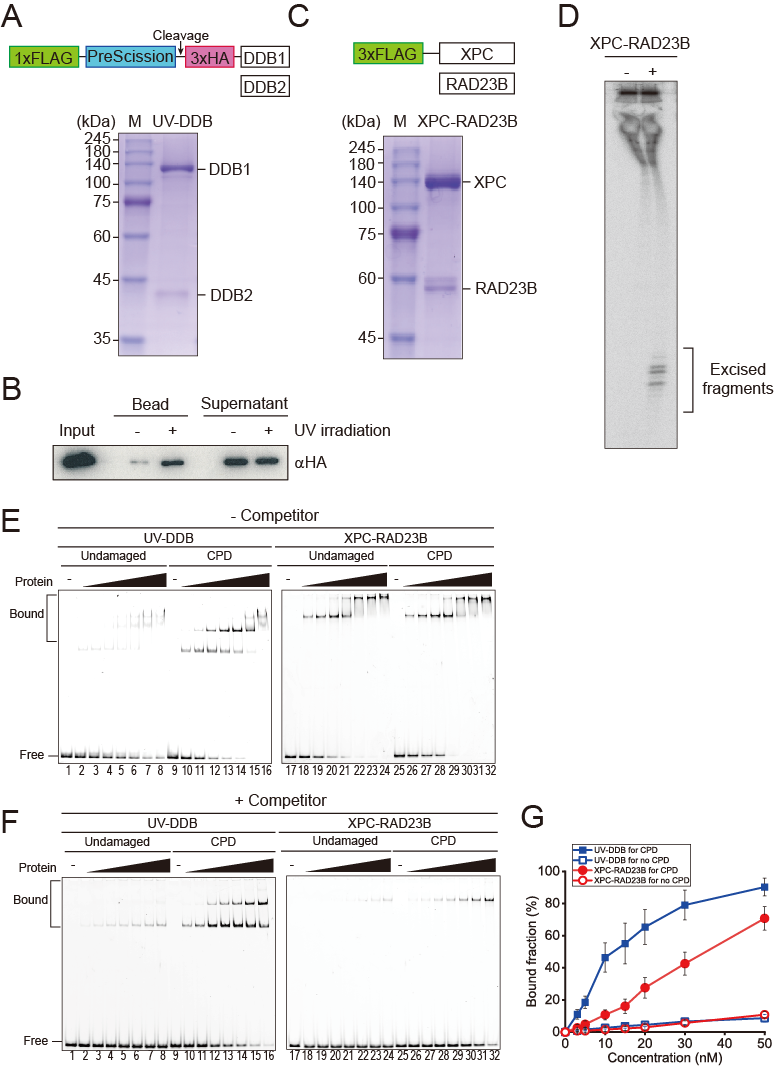


A. (Top) The schematic constructs of DDB1 and DDB2. The amino-terminus of DDB1 has 1xFLAG, PreScission protease cleavage sequence, and 3x HA. 1x FLAG tag was cleaved out by PreScission protease during purification. (Bottom) SDS PAGE gel image of purified UV-DDB.

B. Activity test for the purified UV-DDB, which specifically bound to UV-irradiated DNA molecules that are conjugated with magnetic beads via biotin-streptavidin linkage.

C. (Top) The schematic constructs of XPC-RAD23B. The amino-terminus of XPC has 3xFLAG tag. (Bottom) SDS PAGE gel image of purified XPC-RAD23B

D. *In vitro* NER assay to test the activity of the purified XPC-RAD23B. XP-C cell extracts were mixed with purified XPC-RAD23B. The mixture was incubated with cisplatin-containing plasmid to generate excised DNA fragments by NER, which were radio-labeled by the primer extension method and detected by denaturing PAGE.

E. EMSA for titration of (left) UV-DDB and (right) XPC-RAD23B (0, 3, 5, 10, 15, 20, 30, and 50 nM) to 4 nM undamaged and CPD-containing DNA in the absence of competitors.

F. EMSA for titration of (left) UV-DDB and (right) XPC-RAD23B (0, 3, 5, 10, 15, 20, 30, and 50 nM) to 4 nM undamaged and CPD-containing DNA in the presence of excessive (50x) competitors.

G. Quantification of EMSA in Supplementary Figure S1F. Error bars are obtained from standard error in triplicate.

**Supplementary Figure S2. Single-tethered DNA curtain assay and preparation of CPD-containing lambda DNA**


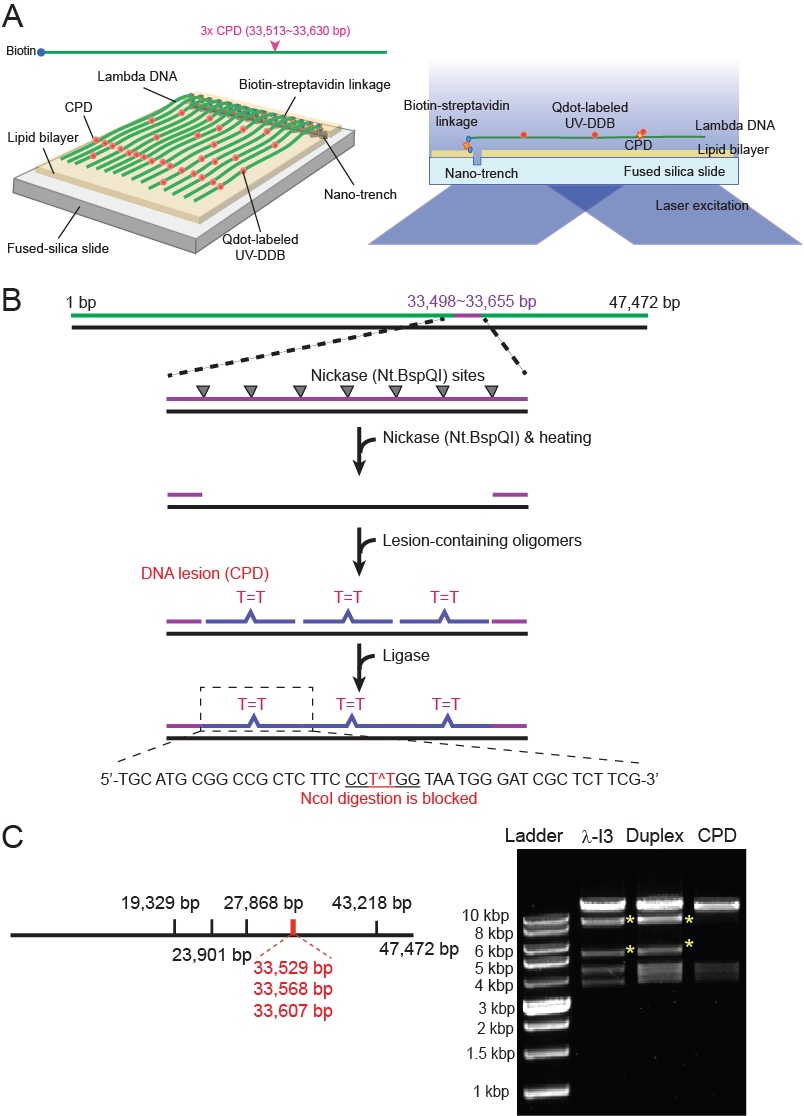


A. Schematic of the single-tethered DNA curtain with nano-trench patterns. (Top) Lambda DNA (λ-DNA) construct containing 3x CPDs. One end of λ-DNA is modified with biotin, and 3x CPDs are placed around 33.5 kbp from the biotinylated end. (Middle) Slant view and (bottom) side view of the DNA curtain. UV-DDB is labeled with anti-HA conjugated Qdot for fluorescence imaging under total internal reflection fluorescence microscopy. Under buffer flow, DNA molecules are stuck at a nano-trench and stretched.

B. Schematic procedure for preparing CPD-containing lambda DNA (λ-DNA) using engineered λ-DNA (λ-I3).

C. (Left) Digestion map of NcoI in λ-I3. (Right) Image of agarose gel electrophoresis for NcoI digestion of λ-I3. Intact, homoduplex-inserted, and CPD-inserted λ-I3 are treated with NcoI. Yellow asterisks indicate the additional cleaved fragments (5,661 bp and 9,611 bp) by NcoI due to the NcoI cognate sites between 33,498 bp and 33,655 bp of λ-I3. For CPD-insertion, there is no such band observed, ensuring that CPD-containing oligomers are well inserted.

**Supplementary Figure S3. Effect of UV-DDB on the XPC-RAD23B binding to CPD**


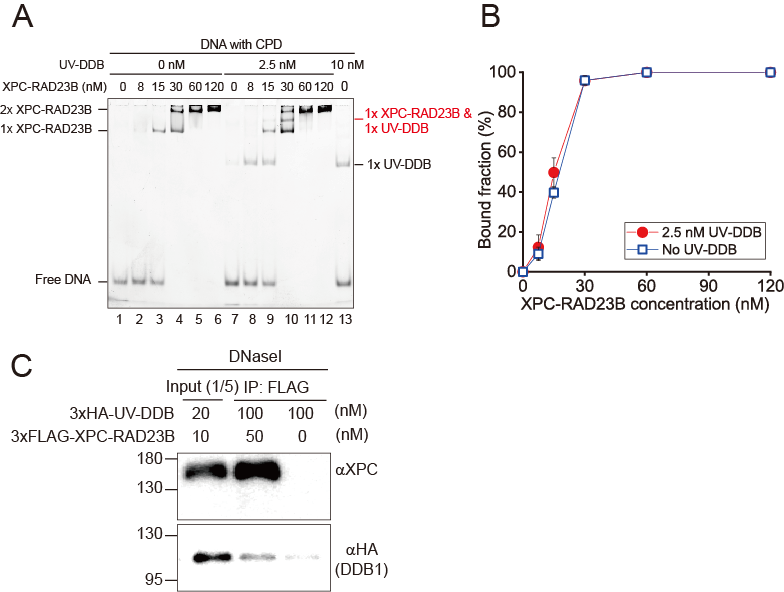


A. EMSA for XPC-RAD23B binding (0, 8, 15, 30, 60, and 120 nM) to CPD-containing DNA in the absence (left) and presence (right) of 2.5 nM UV-DDB. UV-DDB does not enhance the binding of XPC-RAD23B to CPD.

B. Quantification of EMSA in Supplementary Figure S3A. Error bars are obtained from standard error in triplicate.

C. *In vitro* immunoprecipitation (IP) assay for the complex formation of UV-DDB and XPC-RAD23B in the presence of 1 μg/ml DNase I. 3x HA-tagged UV-DDB is pulled down by 3x FLAG-tagged XPC-RAD23B, which is conjugated with anti-FLAG beads. The proteins are blotted by denoted antibodies.

**Supplementary Figure S4. Behavior of UV-DDB on undamaged DNA**


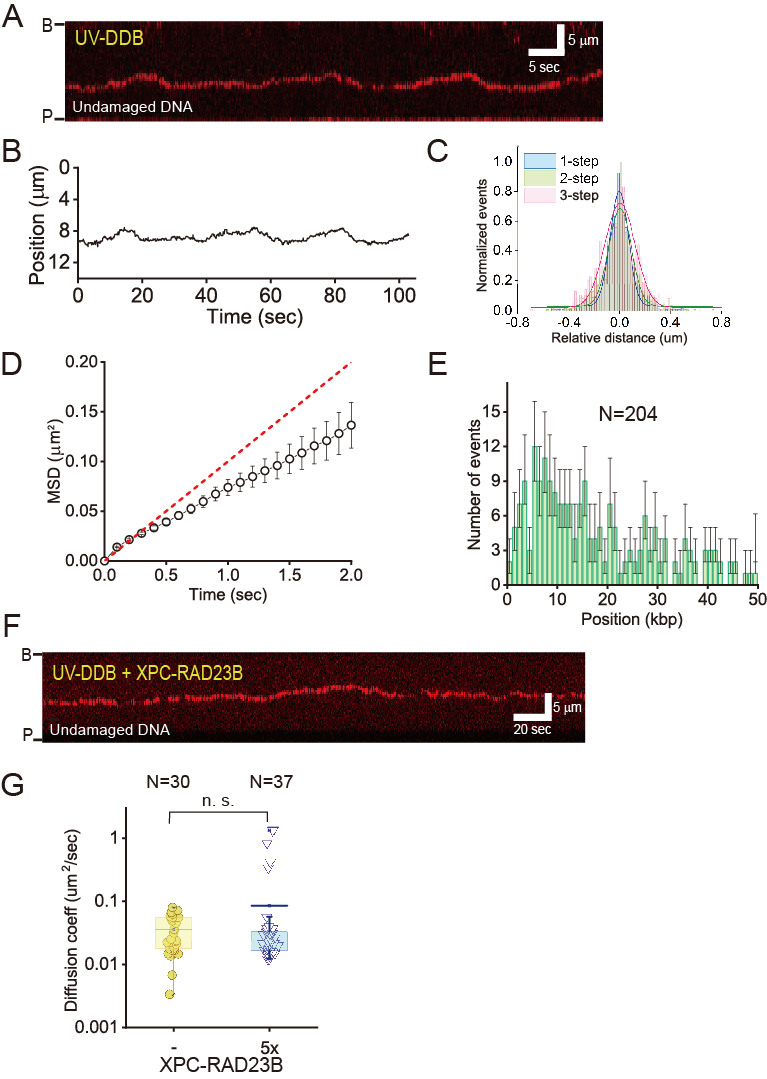


A. Representative kymograph for UV-DDB diffusion on undamaged λ-DNA. B and P at the left indicate barrier and pedestal locations, respectively.

B. Particle tracking result of the kymograph in Supplementary Figure S4A.

C. Distributions of relative displacements from the particle tracking data with (blue) 1-step, (green) 2-step, and (magenta) 3-step. All three distributions are well-fitted with a single Gaussian function having its center at zero, indicating that the particle movement follows Brownian motion.

D. Mean square displacement (open circle) and linear fitting (red dashed line) for diffusion coefficient. The first three data points are fitted to calculate diffusion coefficient.

E. Initial binding distribution of UV-DDB on undamaged DNA in the presence of 5x XPC-RAD23B. N denotes the number of molecules analyzed. The error bars are obtained from bootstrapping with 70% confidence interval.

F. Representative kymograph for UV-DDB diffusion along DNA in the presence of 5x XPC-RAD23B at 50 mM NaCl.

G. Diffusion coefficients of UV-DDB in the presence of 5x XPC-RAD23B at 50 mM NaCl. N denotes the number of molecules analyzed. The diffusion coefficients between the presence and the absence of 5x XPC-RAD23B are not statistically different (n. s.). The p-value of Student’s t-test is 0.17.

**Supplementary Figure S5. Expression of DDB2-mKO1 in U2OS cells**


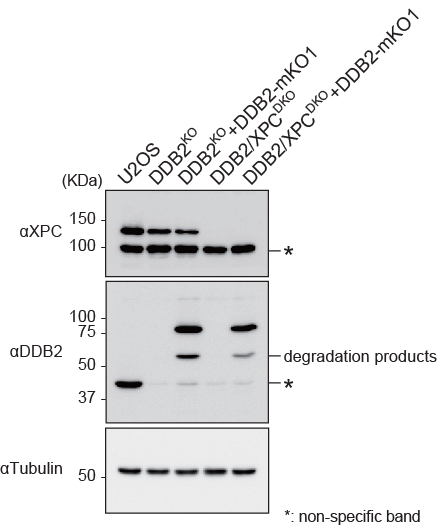


*DDB2* gene is knocked out in U2OS cells, in which *XPC* is either existing (U2OS^DDB2 KO^) or knocked out (U2OS^DDB2/XPC DKO^). Then mKO1-tagged DDB2 (DDB2-mKO1) is expressed in each U2OS cell. The expression of DDB2-mKO1 and XPC is checked by Western blot in each U2OS cell. Each antibody is denoted at the left. The anti-DDB2 antibody used here (R&D Systems, AF3297) cross-reacts non-specifically with the certain protein migrating very close to endogenous DDB2. With these DDB2 KO and DDB2/XPC double KO cell lines, we confirm the absence of wild-type *DDB2* allele by genomic PCR and sequencing. The asterisk (*) indicates nonspecific bands.

**IV. Supplementary References**

1. Cheon, N.Y., Kim, H.S., Yeo, J.E., Schärer, O.D. and Lee, J.Y. (2019) Single-molecule visualization reveals the damage search mechanism for the human NER protein XPC-RAD23B. *Nucleic Acids Res*, **47**, 8337-8347.

2. Sugasawa, K., Okuda, Y., Saijo, M., Nishi, R., Matsuda, N., Chu, G., Mori, T., Iwai, S., Tanaka, K., Tanaka, K. and Hanaoka, F. (2005) UV-induced ubiquitylation of XPC protein mediated by UV-DDB-ubiquitin ligase complex. *Cell*, **121**, 387-400.

3. Dunand-Sauthier, I., Hohl, M., Thorel, F., Jaquier-Gubler, P., Clarkson, S.G. and Schärer, O.D. (2005) The spacer region of XPG mediates recruitment to nucleotide excision repair complexes and determines substrate specificity. *J Biol Chem*, **280**, 7030-7037.

4. Gradia, S.D., Ishida, J.P., Tsai, M.S., Jeans, C., Tainer, J.A. and Fuss, J.O. (2017) MacroBac: New Technologies for Robust and Efficient Large-Scale Production of Recombinant Multiprotein Complexes. *Methods Enzymol*, **592**, 1-26.

5. Kim, M., Kim, H.S., D'Souza, A., Gallagher, K., Jeong, E., Topolska-Wos, A., Ogorodnik Le Meur, K., Tsai, C.L., Tsai, M.S., Kee, M. *et al.* (2022) Two interaction surfaces between XPA and RPA organize the preincision complex in nucleotide excision repair. *Proc Natl Acad Sci U S A*, **119**, e2207408119.

6. Brosey, C.A., Chagot, M.E., Ehrhardt, M., Pretto, D.I., Weiner, B.E. and Chazin, W.J. (2009) NMR analysis of the architecture and functional remodeling of a modular multidomain protein, RPA. *J Am Chem Soc*, **131**, 6346-6347.

7. Hohl, M., Thorel, F., Clarkson, S.G. and Scharer, O.D. (2003) Structural determinants for substrate binding and catalysis by the structure-specific endonuclease XPG. *J Biol Chem*, **278**, 19500-19508.

8. Enzlin, J.H. and Scharer, O.D. (2002) The active site of the DNA repair endonuclease XPF-ERCC1 forms a highly conserved nuclease motif. *EMBO J*, **21**, 2045-2053.

9. Kim, S., Shin, W.H., Kang, Y., Kim, H. and Lee, J.Y. (2024) Direct visualization of replication and R-loop collision using single-molecule imaging. *Nucleic Acids Res*, **52**, 259-273.

10. Kang, Y., Cheon, N.Y., Cha, J., Kim, A., Kim, H.I., Lee, L., Kim, K.O., Jo, K. and Lee, J.Y. (2020) High-throughput single-molecule imaging system using nanofabricated trenches and fluorescent DNA-binding proteins. *Biotechnol Bioeng*, **117**, 1640-1648.

11. Kang, Y., Cheon, N.Y., Cha, J., Kim, A., Kim, H.I., Lee, L., Kim, K.O., Jo, K. and Lee, J.Y. (2020) High-throughput single-molecule imaging system using nanofabricated trenches and fluorescent DNA-binding proteins. *Biotechnol Bioeng*, **117**, 1640-1648.

12. Kim, J., Li, C.L., Chen, X., Cui, Y., Golebiowski, F.M., Wang, H., Hanaoka, F., Sugasawa, K. and Yang, W. (2023) Lesion recognition by XPC, TFIIH and XPA in DNA excision repair. *Nature*, **617**, 170-175.

13. Bagchi, B., Blainey, P.C. and Xie, X.S. (2008) Diffusion constant of a nonspecifically bound protein undergoing curvilinear motion along DNA. *J Phys Chem B*, **112**, 6282-6284.

14. Yeh, J.I., Levine, A.S., Du, S.C., Chinte, U., Ghodke, H., Wang, H., Shi, H.B., Hsieh, C.L., Conway, J.F., Van Houten, B. and Rapic-Otrin, V. (2012) Damaged DNA induced UV-damaged DNA-binding protein (UV-DDB) dimerization and its roles in chromatinized DNA repair. *P Natl Acad Sci USA*, **109**, E2737-E2746.

15. Sakai, W., Yuasa-Sunagawa, M., Kusakabe, M., Kishimoto, A., Matsui, T., Kaneko, Y., Akagi, J.I., Huyghe, N., Ikura, M., Ikura, T. *et al.* (2020) Functional impacts of the ubiquitin-proteasome system on DNA damage recognition in global genome nucleotide excision repair. *Sci Rep*, **10**, 19704.
